# Neural cell adhesion molecule is required for ventricular conduction system development

**DOI:** 10.1242/dev.199431

**Published:** 2021-06-07

**Authors:** Camila Delgado, Lei Bu, Jie Zhang, Fang-Yu Liu, Joseph Sall, Feng-Xia Liang, Andrew J. Furley, Glenn I. Fishman

**Affiliations:** 1 Leon H. Charney Division of Cardiology, Department of Medicine, NYU Grossman School of Medicine, NY 10016, USA; 2 Microscopy Laboratory, Division of Advanced Research Technologies, NYU Langone Health, NY 10016, USA; 3 Department of Biomedical Science, The University of Sheffield, Western Bank, Sheffield, S10 2TN, UK

**Keywords:** Purkinje cell, Cell adhesion molecule, NCAM-1, Polysialic acid, Cardiac conduction system, Ventricular conduction system, Mouse

## Abstract

The most distal portion of the ventricular conduction system (VCS) contains cardiac Purkinje cells (PCs), which are essential for synchronous activation of the ventricular myocardium. Contactin-2 (CNTN2), a member of the immunoglobulin superfamily of cell adhesion molecules (IgSF-CAMs), was previously identified as a marker of the VCS. Through differential transcriptional profiling, we discovered two additional highly enriched IgSF-CAMs in the VCS: NCAM-1 and ALCAM. Immunofluorescence staining showed dynamic expression patterns for each IgSF-CAM during embryonic and early postnatal stages, but ultimately all three proteins became highly enriched in mature PCs. Mice deficient in NCAM-1, but not CNTN2 or ALCAM, exhibited defects in PC gene expression and VCS patterning, as well as cardiac conduction disease. Moreover, using *ST8sia2* and *ST8sia4* knockout mice, we show that inhibition of post-translational modification of NCAM-1 by polysialic acid leads to disrupted trafficking of sarcolemmal intercalated disc proteins to junctional membranes and abnormal expansion of the extracellular space between apposing PCs. Taken together, our data provide insights into the complex developmental biology of the ventricular conduction system.

## Abstract (precis)

**Summary:** The cell adhesion molecule NCAM-1 and its post-translational modification by polysialylation are required for normal formation and function of the specialized ventricular conduction system.

## INTRODUCTION

The cardiac conduction system (CCS) is responsible for tightly regulating the normal rhythmicity of the heart. Comprising several distinct subpopulations of cells with unique functions, including intrinsic automaticity and pacemaker activity in the SA and AV nodes, and rapid impulse conduction in the bundle of His and Purkinje network, each component of the CCS is characterized by a unique transcriptional and functional signature. Although relatively scarce, accounting for less than 1% of all ventricular cardiomyocytes, the Purkinje cells (PCs) within the ventricular conduction system (VCS) have in recent years become the subject of extensive investigation, in large part because there is accumulating experimental and clinical evidence demonstrating that pathologies affecting the PC network, whether developmental or acquired, can increase the risk of arrhythmogenesis and sudden cardiac death (Park and Fishman, 2011).

Leveraging the CCS-*lacZ* reporter mouse (Logan et al., 1993; Rentschler et al., 2001) and differential transcriptional profiling, we previously reported the highly enriched expression of the cell adhesion protein contactin 2 (CNTN2) in the VCS (Pallante et al., 2010). This member of the immunoglobulin superfamily of cell adhesion molecules (IgSF-CAMs) was weakly expressed in the ventricular myocardium in late-stage fetal hearts, but soon after birth its expression was markedly upregulated and highly restricted to PCs within the VCS (Maass et al., 2015). Using *Cntn2*-EGFP BAC transgenic reporter mice, which faithfully recapitulate expression of endogenous *Cntn2* (Heintz, 2004; Pallante et al., 2010), our laboratory subsequently identified a number of additional highly enriched transcripts in PCs, including *ETS* variant transcription factor 1 (ETV1), a key transcriptional regular of the ‘fast conduction’ transcriptome in the atria and VCS (Shekhar et al., 2016), as well as Purkinje cell protein 4 (PCP4), a small IQ motif-containing protein implicated in intracellular calcium homeostasis and PC excitability (Kim et al., 2014).

In the heart, cell adhesion molecules are essential for normal cardiac function, establishing specialized cell-cell contacts that are postulated to be essential for normal electrical coupling and mechanical contraction. With the goal of identifying and characterizing additional cell adhesion proteins in VCS formation and function, in this study we examined differential gene expression datasets comparing postnatal day 21 PCs and working ventricular myocytes (VMs) (Kim et al., 2014; Shekhar et al., 2018). We discovered two additional highly enriched cell adhesion proteins belonging to the IgSF-CAM family: neural cell adhesion molecule 1 (NCAM-1) and activated leukocyte cell adhesion molecule (ALCAM). IgSF-CAMs are a large group of cell adhesion proteins that mediate cell-cell interactions via Ig-like domains and are involved in downstream signaling cascades (von Lersner et al., 2019; Yoshihara, 2009). Through their multifunctionality, IgSF-CAMs play crucial roles in nervous system development (Leshchyns'ka and Sytnyk, 2016; Schmid and Maness, 2008), raising the question of whether members of this family of proteins play a similar role in the CCS and whether there are unique or redundant functional roles for each family member.

Here, using knockout mice for each IgSF-CAM, as well as newly generated *ST8sia2* and *ST8sia4* knockout mice deficient in post-translational sialylation of NCAM-1, we demonstrate a dominant role for NCAM-1 and its post-translational modification by polysialic acid (PSA) (Kudo et al., 1996; von Der Ohe et al., 2002) in PC differentiation and VCS formation and function.

## RESULTS

### Expression of cell adhesion proteins in adult PCs

**Fig. 1.**
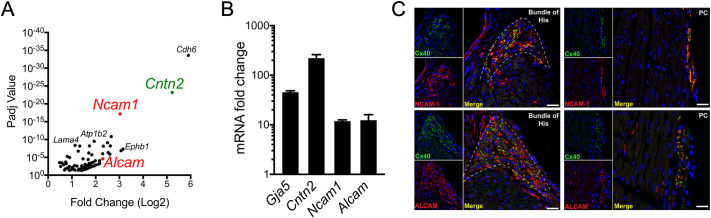
**NCAM-1 and ALCAM are enriched in the murine VCS.** (A) Scatter plots identified *Ncam-1* and *Alcam* (red) as two of the most enriched genes with cell adhesion properties in mature PCs. (B) Quantitative RT-PCR (qPCR) confirmed significant *Ncam-1* and *Alcam* enrichment in adult PCs in addition to other known markers of the VCS (*Cx40* and *Cntn2*) (*n*=3). Error bars represent s.e.m. (C) Immunofluorescence staining in WT P21 murine hearts sections in the VCS shows overlapping expression of NCAM-1 (red) and ALCAM (red) with Cx40 (green) in the HPS. Scale bars: 25 μm.

To identify genes that might influence PC development and VCS function, we analyzed RNA-seq datasets of PCs and VMs isolated by FACS-sorted myocytes prepared from the hearts of postnatal day 21 *Cntn2*-EGFP mice (Shekhar et al., 2018). In addition to the previously described *Cntn2* (Pallante et al., 2010), this analysis revealed several highly enriched cell adhesion molecules in PCs, including two that belong to the immunoglobulin superfamily (IgSF-CAMs): activated leukocyte cell adhesion molecule (*Alcam*) and neural cell adhesion molecule 1 (*Ncam-1*), (Fig. 1A).

Given the co-expression of multiple IgSF-CAMs in PCs, we focused our attention on *Alcam* and *Ncam-1*. Enrichment of both transcripts in PCs was confirmed by quantitative RT- PCR (qPCR), as was known CCS markers [*Cx40* (*Gja5*) and *Cntn2*], validating the *Cntn2-EGFP* FACS-based enrichment strategy for PCs (Fig. 1B). Immunofluorescent staining of adult wild-type (WT) hearts demonstrated robust expression of ALCAM and NCAM-1 specifically in the bundle of His and Purkinje network, with no detectable expression in working VMs. Both ALCAM and NCAM-1 colocalized with Cx40-expressing cells of the VCS and were preferentially detected in the intercalated disc of PCs (Fig. 1C).

### Expression of NCAM-1 and ALCAM in the developing heart

**Fig. 2.**
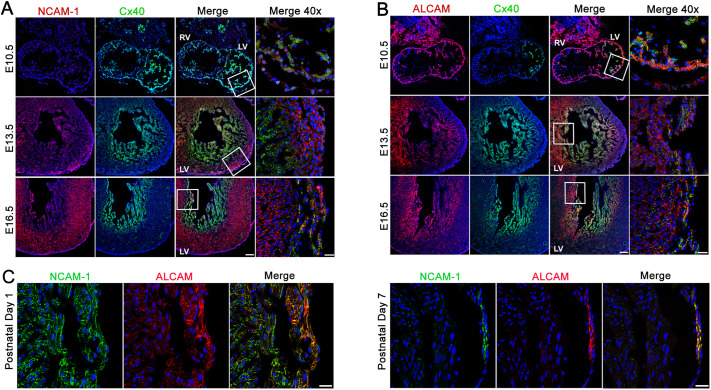
**Spatiotemporal expression of NCAM-1 and ALCAM during myocardial development.** (A) Immunofluorescence staining of NCAM-1 (red) and Cx40 (green) during ventricular murine development at E10.5, E13.5 and E16.5. Right-hand panels show high-magnification views of the boxed areas. NCAM-1 is first detected in the compact myocardium at E13.5. By E16.5, NCAM-1 is expressed in the compact myocardium and a subset of Cx40^+^ cells in the trabecular myocardium. (B) Immunofluorescence staining for ALCAM (red) and Cx40 (green) during ventricular murine development at E10.5, E13.5 and E16.5. Right-hand panels show high-magnification views of the boxed areas. ALCAM is detected as early as E10.5 and its expression remains restricted to the trabecular myocardium. Scale bars: 100 μm (main panels); 25 μm (right-hand panels). (C) Immunofluorescence staining at P1 for NCAM-1 (green) and ALCAM (red) detected expression in trabecular ventricular myocardium, which becomes progressively restricted to the subendocardial region. By P7, NCAM-1 and ALCAM are restricted to PCs and absent from VMs. LV, left ventricle; RV, right ventricle. Scale bars: 25 μm.

To determine the spatiotemporal pattern of expression of ALCAM and NCAM-1 in the developing heart, we performed immunofluorescent staining at sequential stages of embryonic (E10.5, E13.5 and E16.5) and postnatal (P1 and P7) heart developmental. Cx40 expression was used as a marker of trabecular development. At E10.5, NCAM-1 expression was undetectable. By E13.5, NCAM-1 was weakly expressed in the compact myocardium and the interventricular septum (IVS) but absent in the trabecular myocardium. At E16.5, in addition to the compact myocardium, NCAM-1 expression was detected in the subendocardium, colocalizing with Cx40^+^ cells along the IVS and free wall (Fig. 2A).

Expression of ALCAM was detected transmurally as early as E10.5 largely overlapping with Cx40. By E13.5, ALCAM was restricted to the trabecular myocardium and the IVS and this pattern of expression continued for the remainder of embryonic development (Fig. 2B). During the first week of postnatal life, both ALCAM and NCAM-1 became progressively restricted to PCs and by P7 they were no longer detectable in working ventricular myocardium (Fig. 2C).

### Targeted disruption of IgSF-CAMs

To determine the necessity of CNTN2, NCAM-1 and ALCAM in the formation or function of the Purkinje fiber network, we characterized the phenotypic consequences of gene disruption for each individual IgSF-CAM (Cremer et al., 1994; Poliak et al., 2003; Weiner et al., 2004). Homozygous mutant (KO) mice for all three strains were viable and born in expected Mendelian ratios. For *Cntn2* KO and *Alcam* KO mice, heart weights, body weights and heart weight to body weight (HW/BW) ratios were no different than WT littermate controls, whereas heart and body weight were significantly lower in *Ncam-1* KO mice (Fig. S1A). As previously reported (Cremer et al., 1994), *Ncam-1* KO mice were smaller than WT littermates so the HW/BW ratio was not different in these mice, suggesting a proportionate reduction in heart size in *Ncam-1* KO mice (Fig. S1A). Histological assessment revealed no evidence of structural abnormalities or fibrosis in *Alcam*, *Cntn2* or *Ncam-1* KO hearts compared with WT littermates based on Hematoxylin and Eosin or trichrome staining (Fig. S1B). Immunostaining confirmed the absence of ALCAM, CNTN2 or NCAM-1 in PCs of each of the three KO models. Loss of function of individual IgSF-CAMs did not affect trafficking of the gap junction protein Cx40 to the intercalated discs of PCs (Fig. S1C).

**Fig. 3.**
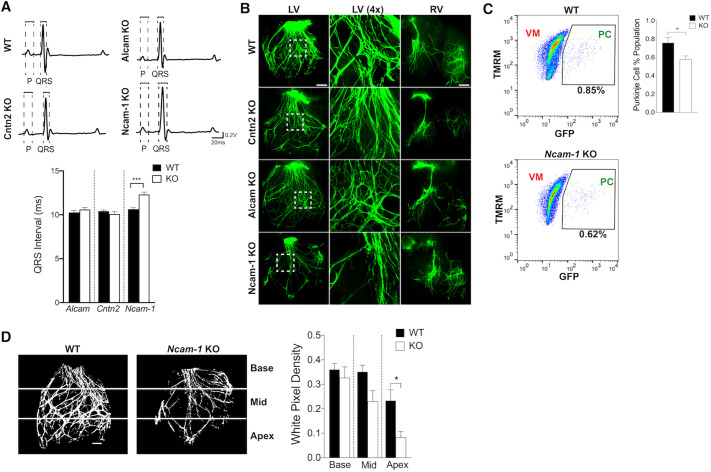
***Ncam-1*-deficient mice display ventricular conduction slowing and a dysmorphic VCS morphology.** (A) Representative surface ECG traces of adult *Cntn2*, *Alcam* and *Ncam-1* WT and KO mice. *Ncam-1* KO mice show a modest but significant prolongation of the QRS interval compared with *Ncam-1* WT controls. No difference in QRS was observed with *Alcam*- or *Cntn2*-deficient mice. (*n*=12 for each genotype). (B) Adult *Cntn2*, *Alcam* and *Ncam-1* KO mice backcrossed into the *Cntn2*-EGFP reporter line were used to visualize the right and left HPS. Center panels are high-magnification views of the boxed areas of the left ventricle (LV). Right ventricle (RV) is displayed in the right-hand panels (*Cntn2* and *Alcam* KO *n*=5; *Ncam-1* KO *n*=9, of which six had overt morphological defects). (C) RFP^+^ cells dyed with TMRM were purified into ventricular (VM) and Purkinje (PC) fractions. Shown are representative FACS plots of PC (RFP^+^GFP^+^) and VM (RFP^+^GFP^−^) populations and the percentages of PCs relative to VMs were calculated for *Ncam-1* WT and KO samples (*n*=4). (D) PC population was also quantified using MATLAB in acquired GFP images from WT and *Ncam-1* KO hearts. Pixel density was calculated per unit of masked region. All comparisons between WT and KO groups were made by applying a two-tailed Student's *t*-test (*n*=4). Data represent mean±s.e.m. *P<0.05, ***P<0.001, WT versus KO. Scale bars: 1 mm.

We next performed functional analyses of *Cntn2* KO, *Alcam* KO and *Ncam-1* KO mice and their respective WT littermate controls. Electrocardiograms were recorded between 8 and 12 weeks of age. Loss of *Cntn2* and *Alcam* did not result in any cardiac conduction abnormalities. In contrast, *Ncam-1* deficient mice displayed QRS prolongation, indicative of abnormal ventricular depolarization (Fig. 3A). Additional parameters, including heart rate, RR interval, P-wave duration and PR interval were comparable between *Ncam-1* deficient mice and their WT littermates (Fig. S2). *Cntn2* KO, *Alcam* KO and *Ncam-1* KO mice had normal cardiac function as assessed by transthoracic echocardiography (Table 1). However, the end diastolic (EDV), end systolic (ESV) and stroke (SV) volume in the *Ncam-1* KO mice were significantly less than in the WT littermates, whereas ejection fraction (EF) was normal, likely a reflection of the proportionally smaller heart size in *Ncam-1* KO mice.

**Table 1.**
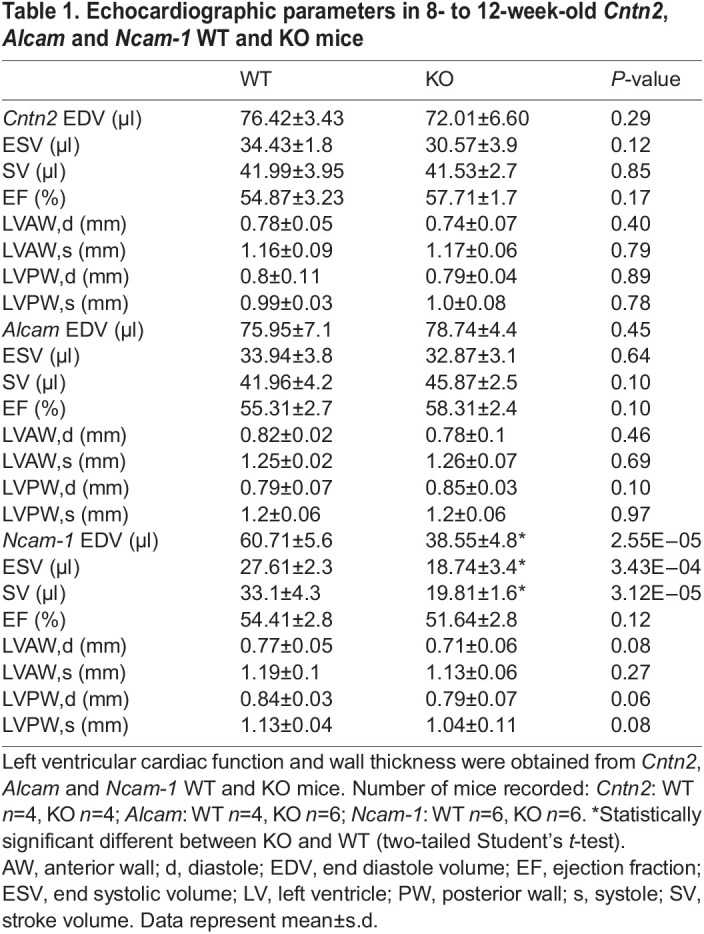
Echocardiographic parameters in 8- to 12-week-old *Cntn2*, *Alcam* and *Ncam-1* WT and KO mice

To determine the mechanistic basis for conduction delay in *Ncam-1* KO mice, we first performed a structural evaluation of the His-Purkinje system (HPS) to assess for morphological defects. All three strains of IgSF-CAM KO mice were backcrossed into the *Cntn2-*EGFP background to facilitate visualization of the HPS by GFP fluorescence. The left and right HPS of *Cntn2* KO and *Alcam* KO mice were comparable to their WT littermates. In contrast, the left HPS in the *Ncam-1* KO showed a highly variable, dysmorphic phenotype, characterized by reduced intercrossing between the fibers in the majority (6/9) of animals (Fig. 3B). Interestingly, no morphological defects were observed in the right HPS of *Ncam-1* KO mice (Fig. 3B). To quantify the percentage of *Cntn2*-EGFP^+^ PCs in WT and KO hearts and determine whether there was a difference in total PC population in the *Ncam-1* KO, ventricles were dissociated into single cells and stained with the red-orange fluorescent mitochondrial marker TMRM to identify cardiomyocytes (Hattori et al., 2010). The percentage of RFP^+^/*Cntn2*-EGFP^+^ PCs relative to total VMs was reduced by 24% in *Ncam-1* KO hearts as assessed by flow cytometry (Fig. 3C). Additionally, GFP signal quantification was performed on WT and *Ncam-1* KO left HPS images. Images were processed using a custom MATLAB algorithm. Each image was divided into three regions: base, mid and apex. The total density for each heart was calculated per unit of masked region. This analysis revealed a 33% reduction in GFP signal in the *Ncam-1* KO hearts localized primarily to the mid and apical regions of the heart (Fig. 3D). To explore whether the decrease in GFP signal in *Ncam-1* KO was due to a defect in proliferation during development, we used the proliferation marker Ki-67 on E15.6 *Ncam-1* WT and KO mice with ALCAM as a trabecular marker. Immunofluorescent staining revealed no proliferation defect in myocytes between *Ncam-1* KO and WT mice (not shown). From these results, we conclude that abnormalities in PC network patterning are likely to contribute to the ventricular conduction slowing observed by electrocardiography.

### Dysregulation of PC gene expression in *Ncam-1* mutant mice

**Fig. 4.**
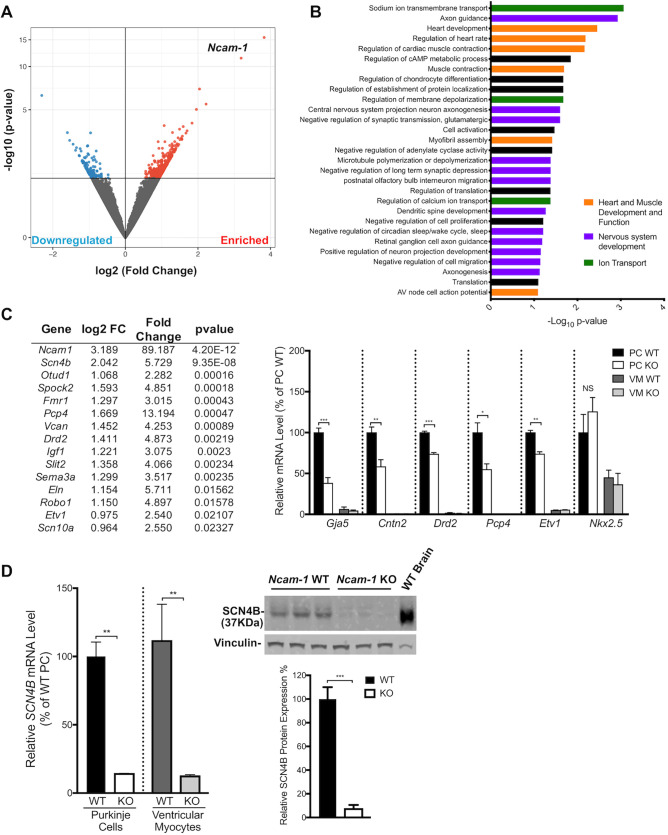
**Dysregulation of PC programming in *Ncam-1* KO mice.** (A) Volcano plot of relative transcript expression from *Ncam-1* WT and KO PCs (*n*=3). All significantly (*P*<0.05) expressed genes are labeled red (enriched) or blue (downregulated) and all nonsignificantly different transcripts are gray. (B) Functional network analysis using the DAVID functional annotation tool of differentially expressed genes from PCs of *Ncam-1* WT and KO mice. (C) Table of selected targets and quantitative RT-PCR of enriched in PCs of *Ncam-1* WT mice compared with PCs of *Ncam-1* KO mice with log2-fold change, fold change and *P*-value. (D) SCN4B in PCs and VMs of *Ncam-1* WT and KO mice (*n*=3). Western blot analysis and quantification of adult brain (30 μg) and heart ventricular lysates (30 μg) probed for SCN4B. Vinculin was used as loading control. Data represent mean±s.e.m. *P<0.05, **P<0.01, ***P <0.001 (two-tailed Student's *t*-test) WT versus KO. NS, not significant.

To identify the molecular mechanisms responsible for the conduction defects and abnormal VCS patterning observed in *Ncam-1* KO mice, we performed RNA-seq transcriptional profiling on adult *Ncam-1* WT and KO mice backcrossed with the *Cntn2-*EGFP reporter line for PC identification. A multitude of transcripts known to be enriched in PCs were confirmed when comparing PCs with VMs isolated from WT hearts, in agreement with previous RNA-seq transcriptional screens previously reported by our laboratory (Shekhar et al., 2018). A comparison of transcriptional profiles in PCs isolated from *Ncam-1* WT versus KO samples revealed 505 differentially expressed protein-coding genes with detectable counts in PCs (normalized counts>5) and *P*<0.05 (Fig. 4A). To classify the gene networks affected by loss of *Ncam-1*, we performed functional network analysis using the DAVID functional annotation tool (Fig. 4B). Among the functional pathways detected, many belong to nervous system development, including axogenesis, neuronal migration, axonal guidance and neuronal projections. Additionally, several pathways previously reported in studies of *Ncam-1* KO neuronal phenotypes were identified, including regulation of circadian rhythm (Shen et al., 1997), retinal ganglion cell axon guidance (Monnier et al., 2001) and olfactory bulb interneuron migration (Cremer et al., 1994; Hu et al., 1996), consistent with known NCAM-1 functions. Importantly, heart and muscle development and function, ion transport and establishment of protein localization were also significantly enriched.

As expected, *Ncam-1* was the top differentially expressed transcript in WT versus KO samples, with no detectable transcript in the *Ncam-1*-deficient PCs. Interestingly, numerous additional PC-enriched transcripts were downregulated in the *Ncam-1* KO PCs (Fig. 4C). qPCR on FACS-purified PCs and VMs from *Ncam-1* WT and KO samples confirmed the downregulation of *Pcp4*, *Drd2* and *Etv1* in PCs from *Ncam-1* KOs whereas no change was observed for *Nkx2-5*, a major transcriptional regulator of cardiac conduction. We also observed downregulation of *Cx40* and *Cntn2* in PCs of *Ncam-1* KO mice, neither of which reached statistical significance in the RNA-seq differential expression analysis (Fig. 4C). Additionally, *Scn4b*, a gene encoding a voltage-gated sodium channel β accessory protein, was found to be significantly downregulated in both PCs and VMs of *Ncam-1* KO hearts compared with their WT counterparts and these findings were confirmed by qPCR (Fig. 4C). Western blots of adult *Ncam-1* WT and KO whole ventricular lysates demonstrated concordant downregulation of SCN4B at the protein level in *Ncam-1*-deficient hearts (Fig. 4D), confirming the validity of the differential transcriptional profiling analysis. From these data, we conclude that, in addition to patterning defects, perturbations in PC electrophysiology may also contribute to conduction slowing in *Ncam-1* KO mice.

### Post-translation polysialylation of NCAM-1 in the developing heart

NCAM-1 function is regulated by post-translational modification by addition of the glycan PSA (Kudo et al., 1996; von Der Ohe et al., 2002) and, interestingly, PSA-deficient mice die prematurely by postnatal week 4 (Weinhold et al., 2005). Given our observations suggesting an important role for NCAM-1 in VCS patterning and function, we next sought to determine whether NCAM-1 polysialylation itself was mechanistically involved. Accordingly, we first determined the developmental profile of NCAM-1 polysialylation in the developing heart by western blot analysis performed at sequential time points from E10.5 to adult stages, with whole brain lysates and *Ncam-1* KO ventricular lysates used as positive and negative controls, respectively. As previously reported (Kurosawa et al., 1997), during mid to late fetal stages, NCAM-1 in the brain has high levels of polysialylation, reflected as a smear on the western blot when probing with antibodies against NCAM-1. After P7, the levels of polysialylation in the brain rapidly decreased, revealing three bands representing the main isoforms of NCAM-1 in the brain: NCAM-180, NCAM-140 and NCAM-120. We confirmed the high levels of polysialylation during mid to late development by probing these same samples with an antibody directed against PSA (Fig. S3).

**Fig. 5.**
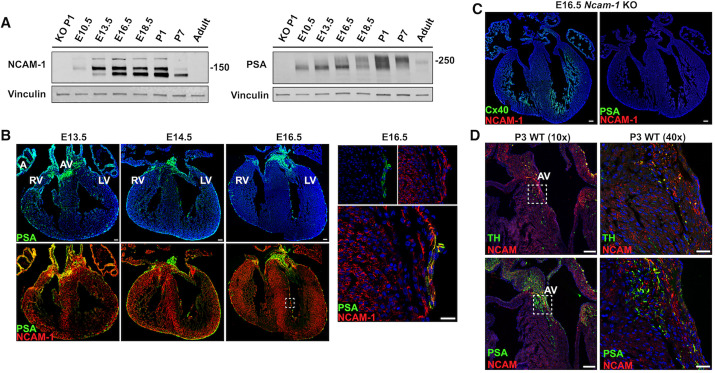
**PSA is expressed in the developing ventricular myocardium.** (A) Western blot analysis of protein extract from whole ventricles at the specified developmental stages using anti-NCAM-1 and anti-PSA-NCAM-1 antibodies. Vinculin was used as loading control. Lysate protein amount loaded: Heart-PSA blot, 15 μg; Heart-NCAM-1 blot, 20 μg. (B) Immunofluorescence staining of NCAM-1 (red) and PSA (green) during ventricular murine development at E13.5, E14.5 and E16.5. PSA is first detected in the atria, AV junction and epicardium at E13.5. By E14.5 and for the remainder of embryonic development, PSA is also detected in NCAM-1^+^ trabecular cardiomyocytes in the subendocardial region. On the right, high-magnification images of the boxed area at E16.5 shows that PSA (green) and NCAM-1 (red) colocalize in the cell membrane. (C) Immunofluorescence staining of E16.5 *Ncam-1* KO sequential sections shows no PSA expression in the absence of NCAM-1. (D) Tyrosine hydroxylase expression in AV junction at P3. Immunofluorescence staining of NCAM-1 (red) with TH (green) and PSA (green) at P3 showing atrioventricular junction region. Right-hand panels show high-magnification views of the boxed areas. AV, atrioventricular junction; LV, left ventricle; RV, right ventricle. Scale bars: 100 μm (B, main panels; C; D, left); 25 μm (B, high-magnification panel; D, right).

Western blots of whole heart lysates using the anti-NCAM-1 antibody detected three bands at 125 kDa, 150 kDa and 200 kDa, indicating the presence of three distinct NCAM-1 isoforms in the developing ventricular myocardium (Fig. 5A). The band at 125 kDa was first detected at E13.5 and gradually increased during late gestation, as previously reported (Wharton et al., 1989). The bands at 150 kDa and 200 kDa bands were detected as early as E10.5, remained present during mid-to-late gestation but rapidly decreased by P7. By P7, the 125 kDa predominated in the early postnatal heart. In the adult working myocardium, NCAM-1 was not detected by western blot, which we attribute to the small population of PCs (<1%) within the adult heart (Boyden et al., 2010). We also probed these same lysates using anti-PSA antibodies and observed a gradual increase of polysialylation in the heart, peaking between E18.5 and P1. The abundance of PSA-modified signal then declined substantially, with little PSA detected in the adult samples (Fig. 5A).

We next evaluated the localization of PSA in the developing heart by immunofluorescent staining at sequential stages of development. PSA was first detected in the atria and atrioventricular (AV) junction at E13.5 with scattered expression in the epicardium. At E14.5, PSA expression was detected in NCAM-1^+^ cells in the subendocardial ventricular region whereas the rest of the NCAM-1^+^ cells in the ventricles remained PSA-free (Fig. 5B). These PSA^+^ cells also expressed Cx40, suggesting they were PC precursors (Fig. 1C). At E16.5 and for the remainder of embryonic development, PSA^+^/NCAM-1^+^ cells continued to be detected from the proximal to distal subendocardial ventricular region. High-magnification imaging of PSA^+^/NCAM-1^+^ cells at E16.5 showed colocalization of PSA and NCAM-1 along the cell membrane (Fig. 5B).

Although NCAM-1 has been shown to be the predominant carrier of PSA in the brain, a small group of additional proteins have been shown to become polysialylated (Hildebrandt et al., 2010). To confirm whether NCAM-1 was also the predominant carrier of PSA in the heart, we performed immunohistochemistry on *Ncam-1* KO hearts at E16.5 with antibodies against PSA. No PSA signal was detected in the *Ncam-1* KO at E16.5 (Fig. 5C), indicating that NCAM-1 is indeed the dominant target for post-translational polysialylation in the murine heart.

Immunostaining at embryonic time points showed strong PSA expression in the AV junction, a region heavily innervated by the autonomic nervous system (Kimura et al., 2012). Because PSA is widely expressed during brain development and is a marker of immature neurons, (Marmur et al., 1998; Seki, 2002) we performed dual staining with tyrosine hydroxylase (TH) antibodies to determine whether the PSA^+^ cells in the AV junction were neuronal in nature. Immunostaining of serial WT heart sections at P3 with anti-NCAM-1 and anti-TH antibodies and with anti-NCAM-1 and anti-PSA antibodies revealed a small percentage of double NCAM-1^+^/TH^+^ cells in the AV junction and bundle of His, indicative of sympathetic neurons (Fig. 5D). Compared with immunostaining on serial sections stained with antibodies against PSA and NCAM-1, the majority of cells that were positive for PSA in the AV junction were negative for TH, suggesting a non-neuronal identity.

### Generation of sialylation-deficient mutant mice

**Fig. 6.**
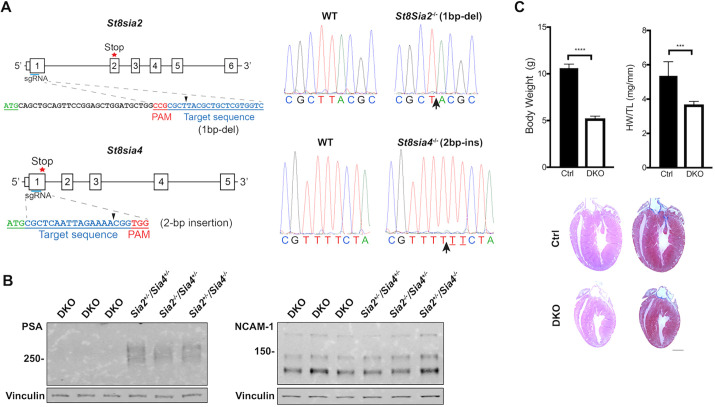
**Generation of PSA-deficient double KO mice by CRISPR/Cas9.** (A) *ST8Sia2* and *ST8Sia4* sgRNA (blue) targeting sequences. Stop codon is labeled with a star and protospacer adjacent motif (PAM) sequence is indicated in red. Black arrowheads indicate the site of the mutation, which is indicated in parentheses for each. Base pair sequence indicates the region where the mutation is, which is indicated with an arrow in the KO sequences by Sanger sequencing. (B) Western blot of protein extract from whole ventricles from PSA-deficient DKOs and littermate controls at P1 (10 μg). Results show that PSA is not detected in DKO mice but is still detected in *Sia2^+/−^/Sia4^+/−^*, *Sia2^−/−^/Sia4^+/−^* and *Sia2^+/−^/Sia4^−/−^* littermates. Probing with anti-NCAM-1 antibody reveals unaffected NCAM-1 levels for all heart samples. Vinculin was used as loading control. (C) Body weight and heart weight/tibia length (HR/TL) ratios of Ctrl (DHet) and PSA-deficient DKO hearts. Hematoxylin and Eosin and trichrome stain of Ctrl (DHet) and PSA-deficient DKO hearts is shown below. Data represent mean±s.e.m. ***P<0.001, ****P<0.0001 (two-tailed Student's *t*-test) of Ctrl (DHet) versus DKO. Scale bar: 1 mm.

To examine the specific role of PSA modification of NCAM-1 in the formation and function of the VCS, we generated PSA-deficient *ST8Sia2/ST8Sia4* double knockout mice (DKO) using CRISPR/Cas9 technology*. ST8Sia2* and *ST8Sia4* are the two polysialyltransferases (polySTs) that synthesize PSA on NCAM-1 (Mühlenhoff et al., 2013; Nakayama et al., 1998) and mice lacking both polySTs have been shown to be deficient in PSA (Weinhold et al., 2005). Sequences within exon 1 of each gene were selected for targeting (Fig. 6A) and founders with frame shifts and early termination within each of the two genes were selected for expansion and phenotypic characterization (Table S2). Breeding pairs of *ST8Sia2^+/−^ST8Sia4^−/−^* (Het/KO)×*ST8Sia2^−/−^ST8Sia4^+/−^* (KO/Het) mice were set up in order to obtain *ST8Sia2^−/−^ST8Sia4^−/−^* DKO PSA-deficient offspring. Sanger sequencing with *ST8Sia2* and *ST8Sia4* specific primers (Table S3) was used to confirm homozygous knockout mutations for both genes (Fig. 6A). Absence of PSA in DKO mice was confirmed by western blot analysis while still being detected in all other littermates. Western blot analysis using anti-NCAM-1 antibodies also confirmed that NCAM-1 expression was preserved in all genotypes, including the DKO mice (Fig. 6B).

PSA-deficient DKO mice were significantly smaller than their littermates and displayed the gross phenotypic abnormalities described previously, including cachexia after 3 weeks, reduced fatty tissue and muscle mass; fewer than 20% of PSA-deficient DKO mice survived past week 4 (Weinhold et al., 2005). Owing to the cachexia and decrease in muscle mass and fatty tissue in DKO mice, we compared heart weight (HW) to tibial length (TL) instead of to body weight (BW), to have a more accurate evaluation of heart size. PSA-deficient DKO mice displayed a reduced HW even when normalized to TL (Fig. 6C). Hematoxylin and Eosin or trichrome staining did not reveal any gross abnormalities or abnormal fibrosis in DKO hearts compared with control double heterozygous littermates (Fig. 6C).

### PSA deficiency results in mislocalization of PC-specific proteins

**Fig. 7.**
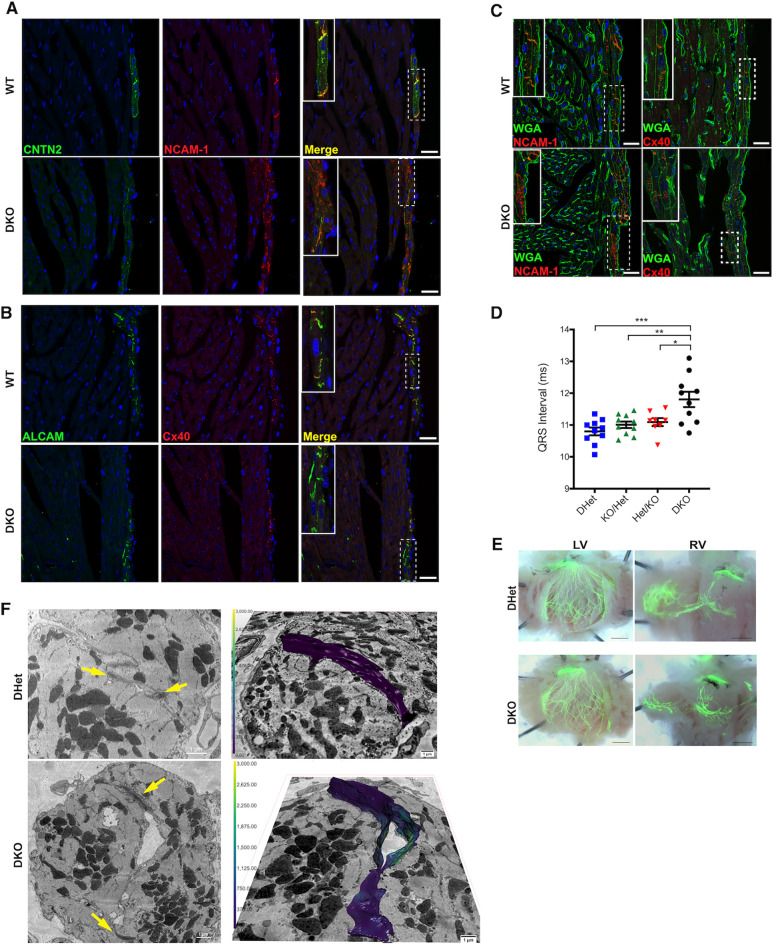
**Mislocalization of cell membrane proteins of PCs in PSA-deficient mice.** (A) Immunofluorescence staining of P21 WT and PSA-deficient hearts sections with antibodies against NCAM-1 (red) and CNTN2 (green). In WT sections, PCs show colocalization of CNTN2 and NCAM-1 in the intercalated disc. PSA-deficient mice show nonoverlapping regions of CNTN2 and NCAM-1. Insets are magnified images of the dashed boxed regions. (B) Immunofluorescence staining of PCs of WT and PSA-deficient P21 hearts with antibodies against Cx40 (red) and ALCAM (green). Insets are magnified images of the dashed boxed regions showing ALCAM^+^ PCs that are negative for Cx40. (C) Immunofluorescence staining shows incomplete localization of Cx40 or NCAM-1 (red) to the cell membrane delineated by WGA (green) in DKO PSA-deficient murine hearts at P21. Insets are magnified images of dashed boxed regions. (D) ECGs of PSA-deficient DKO (*n*=10) mice and littermates with allelic combinations: DHet (*n*=10), Het/KO (*n*=10) and KO/Het (*n*=8). PSA-deficient DKO mice displayed prolonged QRS duration compared with all three other allelic variants. Data represent mean±s.e.m. *P<0.05, **P<0.01, ***P<0.0001 (one-way ANOVA). (E) Left and right ventricle whole-mount dissections of DHet and PSA-deficient DKO mice in the Cntn2-EGFP background. LV, left ventricle; RV, right ventricle. (F) Representative images of 3D SEM sections of DKO and DHet P21 heart sections. Yellow arrows point to cell junctions. Color scale indicates the thickness of the three-dimensional mesh. The colors ranging from purple to yellow represent narrower to wider volumes between membranes, respectively. Scale bars: 25 μm (A-C); 1 mm (E); 1 μm (F).

We next investigated whether the absence of PSA on NCAM-1 influenced the localization of sarcolemmal proteins in PCs. Indeed, in contrast to P21 WT hearts, where CNTN2 and NCAM-1 both localized to the lateral membrane and intercalated discs (IDs) of PCs, in the DKO hearts we observed both diminished abundance of CNTN2 and, even more strikingly, CNTN2 and NCAM-1 no longer colocalized to the ID (Fig. 7A). Moreover, whereas in WT hearts Cx40 and ALCAM colocalized within PCs in the ID, in the KO hearts we observed loss of Cx40 in ALCAM^+^ IDs, suggesting mislocalization of Cx40 as well (Fig. 7B).

Double staining of WT hearts with wheat-germ agglutinin (WGA) and NCAM-1 or WGA and Cx40 also demonstrated sarcolemmal colocalization. In contrast, in the DKO mice there was obvious re-distribution of NCAM-1 and Cx40 away from the membrane and into the cytoplasmic compartment (Fig. 7C). Additional abnormalities were observed when double staining for CNTN2 and plakoglobin, a component of both desmosomes and adherens junctions in cardiomyocytes. Although plakoglobin and CNTN2 colocalized in the ID of WT PCs, the degree of overlap was markedly reduced in the DKO mice (Fig. S4). Taken together, these data suggest an essential role for post-translational polysialylation of NCAM-1 in the targeting of multiple sarcolemmal proteins to the ID in PCs.

### Cardiac conduction abnormalities in PSA-deficient mice

To determine whether loss of PSA, despite preserved expression of NCAM-1, resulted in conduction abnormalities, we performed electrophysiological analysis in DKO PSA-deficient mice and their littermates. The genotypes tested were: *ST8Sia2^−/−^ST8Sia4^−/−^* (DKO), *ST8Sia2^−/+^ST8Sia4^−/+^* (DHet), *ST8Sia2^−/+−^ST8Sia4^−/−^* (Het/KO) and *ST8Sia2^−/−^ST8Sia4^−^/^+^* (KO/Het). Electrocardiograms revealed no change in HR, or RR and PR intervals among the strains (Fig. S5); however, DKO exhibited prolonged QRS durations on electrocardiograms (ECGs) compared with all three other genotypes (Fig. 7D). We also visualized the HPS using the *Cntn2*-EGFP reporter background. Unlike the *Ncam-1* KO mice, this analysis revealed no gross abnormalities in the left or right HPS in any of the genotypes, including the DKO hearts (Fig. 7E). Taken together, these data suggest that failure to post-translationally modify NCAM-1 by polysialylation leads to conduction defects, at least in part through mislocalization of sarcolemmal proteins and disruption of intercellular junctions.

### Ultrastructural PC defects in PSA-deficient mice

To provide additional insight into potential defects in cell junctions in PSA-deficient hearts and determine the topology of the intercellular space between adjacent PCs at high resolution, we performed three-dimensional electron microscopy (3D SEM) by serial block face scanning electron microscopy (SBF SEM) using hearts isolated from P21 DKO mice and DHet littermates. PCs were identified as the first cell layer underlying the ventricular endocardium and by their relatively high glycogen content, as previously described in canine and sheep PCs (Muir, 1957; Sommer and Johnson, 1968; Thornell and Eriksson, 1981). High glycogen content in murine PCs was confirmed via Periodic Acid-Schiff (PAS) staining (Fig. S6). Through *z*-stack acquisition and segmentation, we observed significant abnormal expansion of the extracellular space between the cell membranes of apposing PCs in DKO hearts compared with control mouse hearts (Fig. 7F, Movies 1-6). This finding was suggestive of disruption of cell adhesion and consistent with the defects in trafficking of junctional proteins described above.

## DISCUSSION

In this study, using a fluorescently tagged *Cntn2* reporter gene as a probe for cardiac PCs, we identified and characterized two previously unknown IgSF-CAMs in the adult VCS: NCAM-1 and ALCAM. Using a loss-of-function strategy for each of these three IgSF-CAMs, we demonstrate that NCAM-1, but not CNTN2 or ALCAM, is essential for normal development of the VCS. In its absence, not only is the patterning of the VCS disrupted, but the transcriptional signature of individual PCs within the VCS is perturbed. Indeed, transcriptional profiling revealed downregulation of hundreds of genes, including many that are essential for normal PC electrophysiology, such as transcription factors, ion channel subunits and regulatory proteins. We also show that post-translational polysialylation of NCAM-1 in the developing VCS is required for preserving the ultrastructural integrity of the VCS, as genetic inactivation of *ST8Sia2* and *ST8Sia4* results in mistargeting of key sarcolemmal proteins away from the intercalated disc and expansion of the extracellular space between apposing PCs.

Through immunofluorescence techniques, we first demonstrate that ALCAM and NCAM-1 were enriched in adult PCs but undetectable in VMs at the transcriptional and protein level. We then explored the spatiotemporal patterns of expression for NCAM-1 and ALCAM in the developing myocardium. The unique pattern of expression of NCAM-1 during embryonic development, specifically its restriction to PCs by P7 raises some intriguing questions regarding how NCAM-1 is developmentally regulated in the heart. In the nervous system, three main isoforms of NCAM-1 have been described – NCAM-180, NCAM-140 and NCAM-120 – with the main difference among them being localization to the transmembrane and cytoplasmic domains (Cunningham et al., 1987). Interestingly, differences have been noted among the isoforms in their temporal expression, cell type localization and signaling pathways they are involved in (Beggs et al., 1997; Cox et al., 2009; Cunningham et al., 1987; Noble et al., 1985). These distinct features among NCAM-1 isoforms suggest that in the developing murine myocardium, the three isoforms detected could also display preferential regional localization that respond to different developmental cues. Future experiments will focus on characterizing the localization of the isoforms found in the developing ventricles (125 kDa, 150 kDa and 200 kDa), and their individual roles in cardiac development. Furthermore, detection of the PSA modification only in subendocardial NCAM-1^+^ cells of the developing VCS but not in the compact layer suggests additional differences in regulatory mechanisms between NCAM-1^+^ cells in these two regions.

We observed no overt electrophysiological abnormalities or morphological defects with genetic inactivation of either *Cntn2* or *Alcam*, suggesting that some degree of functional redundancy might be at play in the context of VCS development. In neuronal cells it has been shown that not until multiple members of the IgSF-CAM subfamily are deleted is there a demonstrable mutant phenotype, consistent with our observation in the VCS (Ko et al., 2011; Sakurai et al., 2001). In contrast, multiple abnormalities were detected in *Ncam-1* KO mice, including ventricular conduction delay, a dysmorphic VCS and a decrease in the number of GFP^+^ PCs. Interestingly, the abnormal patterning of the VCS appeared most prominent in the mid and apical aspects of the left HPS. Whether this sidedness is a consequence of generalized left-right embryonic patterning or is more restricted to heart development remains to be determined. For example, it is conceivable that differential contribution of first and second heart field precursors to the VCS within the left and right ventricular chambers influence their sensitivity to *Ncam-1* deficiency (Mohan et al., 2017). In this regard, it is noteworthy that transcriptional profiling studies demonstrate both proximal to distal and left versus right differences in gene expression in the developing and mature VCS (Atkinson et al., 2011; Goodyer et al., 2019). Although our PC isolation strategy did not distinguish chamber of origin, our global transcriptional profiling identified dysregulated expression of multiple PC markers in *Ncam-1*-deficient hearts, suggesting that this cell adhesion protein is essential for normal PC differentiation. Future studies will be required to define more fully the precise mechanisms through which this developmental modulation is orchestrated. NCAM-1 is known to activate several downstream signaling cascades that may impact transcriptional output. These include MAP-kinase pathway activation through interaction with both FGFR and Fyn/FAK, (Kiselyov et al., 2005; Kolkova et al., 2000; Schmid et al., 1999) and activation of FGFR by NCAM-1, thereby influencing cytoplasmic Ca^2+^ concentrations (Doherty et al., 1991; Williams et al., 1994). Interestingly, our lab recently reported that ETV1, a transcription factor crucial for fast conduction programming in the heart, is induced through activation of the Ras-MAPK pathway (Shekhar et al., 2016). Our RNA-seq analysis of *Ncam-1* WT versus KO PCs showed *Etv1* as one of the targets downregulated with the loss of *Ncam-1*. Further exploration of whether NCAM-1 acts through the Ras-MAPK signaling cascade could elucidate its mechanism of action in PC maturation. Finally, given the patterning abnormalities observed in the left His-Purkinje network of *Ncam-1* KO mice, it is conceivable that electromechanical dyssynchrony may promote transcriptional remodeling in PCs, a phenomenon that has been well characterized in dyssynchronous working ventricular myocardium (Barth et al., 2009).

An additional interesting finding was the dramatic downregulation of *Scn4b* in both ventricular VMs and PCs of *Ncam-1* KO mice. The RNA-seq analysis was performed in adult *Ncam-1* WT and KO, when NCAM-1 is undetectable in VMs. Nevertheless, SCN4B mRNA and protein levels were significantly downregulated in both PCs and VMs in adult *Ncam-1* KO mice compared with WT. This opens up the question of the lasting effects into adulthood after loss of *Ncam-1* during myocardial development. SCN4B is one of four auxiliary β subunits that associates with the pore-forming α subunits of voltage-gated sodium channels to modify channel function and mediate protein-protein interactions modulating cell adhesion and migration (Brackenbury and Isom, 2011). SCN4B is known to be expressed in the intercalated discs of cardiomyocytes colocalizing with Cx43 (Gja1) and Nav1.5 (Scn5a) (Maier et al., 2004) and mutations in *SCN4B* have been linked with sudden infant death (Tan et al., 2010). Given these observations, it will be of interest to determine whether abnormalities in inward sodium currents are detected in PCs isolated from NCAM-1 mutant mice.

Finally, we demonstrate the post-translational addition of PSA in a subset of NCAM-1^+^ cells during ventricular development. We show that PSA is first detected in this population of cells at E14.5 with downregulation occurring postnatally. Interestingly, this is reminiscent of the dynamics of PSA expression described during neurodevelopment (Hildebrandt et al., 2007; Kurosawa et al., 1997). Our results are also consistent with previous studies showing expression of NCAM-1 across the myocardium of chick embryos, but restricted expression of PSA-NCAM localized to the atrioventricular canal and ventricular trabeculae (Watanabe et al., 1992). Taken together, our data suggest a conserved developmental role for PSA modification of NCAM-1 in VCS development.

The initial detection of PSA-NCAM in mid to late gestation and its rapid postnatal decline coincides with several morphological changes that are observed in cardiomyocytes during these developmental stages. These include changes in cellular morphology, cell-cell contact formation and distribution of the extracellular matrix. At E14.5, when we first detect PSA in the ventricles, cardiomyocytes are transitioning from a rounded, isotropic morphology to a more polarized ‘brick-like’ shape, characteristic of adult VMs. Interestingly, even though the cells begins to elongate at E14.5, the main cell-cell contact proteins of the future ID, including those that comprise adherens junctions, desmosomes and gap junctions, are all still expressed circumferentially within the sarcolemma at this stage, and it is not until E18.5-P7 that ID proteins become increasingly restricted to ends of the polarizing cells (Angst et al., 1997; Fromaget et al., 1992; Hirschy et al., 2006). These observations suggest that the perinatal period is a critical window of time for proper localization and stabilization of sarcolemmal proteins involved in cell-cell interactions, not only within the working myocardium but also within the developing VCS.

The post-translational modification of NCAM-1 by polysialylation adds an additional layer of complexity to deciphering the role of this protein in VCS development. We tackled this problem by generating PSA-deficient *ST8Sia2/ST8Sia4* double knockout mice. The polysialyltransferases *ST8Sia2* and *ST8Sia4* are responsible for synthesizing PSA on NCAM-1 and genetic deletion of the two genes results in a PSA-deficient mouse (Mühlenhoff et al., 2013; Weinhold et al., 2005). Immunostaining of PSA-deficient mice revealed profound mislocalization of several PC-enriched proteins that are normally targeted to the intercalated disc in the adult VCS, including NCAM-1, CNTN2 and Cx40. These results suggest that disruption of PSA modification on NCAM-1 has an impact on protein trafficking and/or stabilization within discrete subcellular compartments within the sarcolemma, and, remarkably, the defect persists well beyond the developmental window during which PSA is transiently expressed. Mechanistically, we propose that the preferential expression of PSA only on NCAM-1 in the lateral sarcolemmal membrane inhibits cell-cell interactions in that domain, whereas its absence at the ID, in concert with robust expression of unmodified NCAM-1, CNTN2 and ALCAM, is permissive for strong cell-cell interactions. These observations indicate that expression of both native and PSA-modified NCAM-1 are essential for establishing the ramifying cable-like structure that defines the VCS.

Interestingly, this mis-targeting phenotype is not observed in *Ncam-1* mutant mice. This difference may reflect the disparate consequences of altering primarily cell adhesion (*ST8Sia2/ST8Sia4* KO alone), compared with also perturbing NCAM-1-dependent cell signaling cascades (*Ncam-1* KO). However, this scheme is almost certainly an oversimplification, as PSA has been shown to be a negative regulator of NCAM-1 signaling. Removal of PSA resulted in FGFR activation leading to ERK1/2 promotion of differentiation and reduced proliferation (Eggers et al., 2011; Seidenfaden et al., 2003). Alternatively, the phenotype observed in PCs could be PSA specific but NCAM-1 independent through regulation of neighboring molecules affected by the loss of PSA. To examine the ultrastructural consequences of PSA deficiency in the VCS, particularly at cell junctions, we employed transmission 3D SEM. These studies revealed large intercellular spaces in the PSA-deficient double KO mice that were not evident in control PCs. A limitation of this technique is the low number of PCs per section analyzed that are in the appropriate orientation due to their localization in the first layer under the endocardium.

Finally, it is worth noting that our transcriptional screen identified several additional PC-enriched transcripts encoding cell adhesion molecules, such as *Cdh6*. This gene encodes cadherin 6, a membrane glycoprotein that mediates homophilic cell-cell adhesion (Shimoyama et al., 1995). Expression of *Cdh6* has not previously been described in the conduction system, but, interestingly, like numerous PC-enriched transcripts, it has been best characterized in neuronal cell lineages, including neural crest cells (Ishii et al., 2005), and therefore its further characterization in the VCS will likely be of interest.

In summary, we have identified and characterized two previously unknown IgSF-CAMs in the VCS and demonstrated that NCAM-1 deficiency leads to defects in PC gene expression, VCS patterning and cardiac conduction disease. We also show that post-translational modification of NCAM-1 by addition of PSA is restricted in time and space to a small population of late fetal and early perinatal subendocardial cardiomyocytes that ultimately comprise the specialized VCS. Distinct from the phenotype of NCAM-1 deficiency, inhibition of NCAM-1 polysialylation by targeted inactivation of *ST8sia2* and *ST8sia4* results in mislocalization of intercalated disc proteins from the junctional membrane and abnormal expansion of the extracellular space between apposing PCs. Taken together, our data provide new insights into the complex developmental biology of the specialized VCS.

## MATERIALS AND METHODS

### Mutant mice

*Cntn2-*EGFP BAC transgenic (Pallante et al., 2010), *Tag-1^−/−^* (*Cntn2^−/−^*) (Poliak et al., 2003), *Ncam-1^−/−^* (Cremer et al., 1994) and *BEN^−/−^* (*Alcam^−/−^*) (Weiner et al., 2004) mutant mice have all been previously described. *Cntn2-*EGFP BAC transgenic was maintained in a CD1 genetic background. *Ncam-1^−/−^* and *Alcam^−/−^* were maintained in a C57BL/6 background and *Cntn2^−/−^* was maintained in a mixed CD1/C57BL/6 background. For PC FACS and HPS morphology imaging and quantification the *Cntn2^−/−^*, *Ncam-1^−/−^* and *Alcam^−/−^* mouse lines were crossed with *Cntn2-EGFP* mice.

### Generation of CRISPR mice

We followed a standard protocol for generation of knockout mice using the CRISPR/Cas9 system as described (Harms et al., 2014). In brief, the online tools (http://crispor.tefor.net/ or http://crispr.mit.edu) were used to design single guide RNAs (sgRNAs) targeting specific sequences around the start codon of *St8Sia2* and *St8Sia4* (*St8Sia2* sgRNA: GACCACGAGCAGCGTAAGCG; *St8Sia4* sgRNA: GCGCTCAATTAGAAAACGG). Both sgRNAs were cloned into px458 (Addgene) using the restriction endonuclease BbsI. The oligos containing T7 promoter sequence (*St8Sia2 T7* sgRNA forward: TTAATACGACTCACTATAGGGACCACGAGCAGCGTAAGCG; *St8Sia4 T7* sgRNA forward: TTAATACGACTCACTATAGGGCGCTCA ATTAGAAAACGG) and the linearized px458-sgRNA plasmids were then used for preparation of the sgRNA template DNAs for RNA transcription. sgRNAs were synthesized using a MEGAshortscript T7 Transcription Kit (Invitrogen) and purified using a MegaClear Kit (Ambion). The purified sgRNAs along with Cas9 mRNA (TriLink, L7206) were used for microinjections of fertilized eggs of C57BL/6J females with sperm from *Cntn2-EGFP* males. Embryos were transferred into ten recipient pseudopregnant C57BL/6J females and 70 founder pups were born. Founders were genotyped to determine mutations by Sanger sequencing of genomic DNA with primers for *St8Sia2* and *St8Sia4* (Table S3) and TA cloning (Thermo Fisher Scientific, 450030) was used to confirm each allele mutation. Two founders (founder 5 and 11) were chosen for breeding. Each founder was maintained in a C57BL/6 background. For HPS morphology imaging and quantification, the *St8Sia2*/*St8Sia4* mouse lines were crossed with *Cntn2-EGFP* mice.

### Antibody reagents

#### Immunofluorescence

Primary antibodies used were: NCAM-1 (1:100, rabbit, Millipore, ab5032; rat, Millipore, mab310); ALCAM (1:50, goat, R&D Systems, AF1172); CNTN2 (1:50, goat, R&D Systems, AF4439); Cx40 (1:500, rabbit, Alpha Diagnostic, Cx40A); PSA (1:500, rabbit, Absolute Antibody, Ab00240-23.0); troponin T (1:100, mouse, Thermo Fisher Scientific MS-295-P0); plakoglobin (1:100, rabbit, Cell Signaling Technology, 2309S). Secondary antibodies used were: donkey anti-rabbit 555 (1:500, Thermo Fisher Scientific, A31572); donkey anti-goat 555 (1:500, Thermo Fisher Scientific, A21432); goat anti-rat 555 (1:500, Thermo Fisher Scientific, A21434); goat anti-rabbit 488 (1:500, Thermo Fisher Scientific, A11034); donkey anti-rabbit 488 (1:500, Thermo Fisher Scientific, A21206); donkey anti-goat 488 (1:500, Thermo Fisher Scientific, A11055); donkey anti-mouse 488 (1:500, Thermo Fisher Scientific, A21202); donkey anti-rat 488 (1:500, Thermo Fisher Scientific, A21208); wheat germ agglutinin (WGA) Alexa Fluor 488 conjugate (1:250, Thermo Fisher Scientific, W11261).

#### Western blotting

Primary antibodies used were: NCAM-1 (1:400, rabbit, Alomone, ANR-041); PSA (1:500, rabbit, Absolute Antibody, Ab00240-23.0); SCN4B (1:1000, rabbit, Abcam, ab80539); vinculin (1:10,000, mouse Abcam, ab130007). Secondary antibodies used were: goat anti-rabbit (1:15,000, LI-COR, 926-32211); goat anti-mouse (1:15,000, LI-COR, 926-68070).

### Immunohistochemistry

Adolescent and adult hearts were excised and immediately Langendorff perfusion-fixed in 4% cold paraformaldehyde (PFA) overnight. P1-P7 hearts were excised and immediately fixed in cold 4% PFA overnight. Embryonic hearts were excised and immediately fixed in cold 4% PFA for 2 h. Samples were then washed three times in cold 1× PBS and equilibrated in a 30% sucrose solution at 4°C overnight. The samples were then embedded into Tissue-Tek OCT compound (Thermo Fisher Scientific) and frozen. Frozen tissues were cut (10-μm-thick sections) on Superfrost Plus microscope slides (Thermo Fisher Scientific) and stored at −80°C. All sections were blocked with 10% donkey serum and 0.01% Triton X-100 in PBS for 1 h followed by overnight incubation with antibodies at 4°C. Sections were then washed in 1× PBS and incubated with Alexa Fluor dye-conjugated secondary antibodies for 1 h followed by three washes with 1× PBS. Slides were coverslipped with Vectashield mounting media with DAPI (Vector Laboratories). Stained sections were visualized with a Leica TCS SP5 confocal microscope using Leica LAS AF acquisition software.

### Cardiomyocyte enzymatic dissociation for FACS purification

Ventricular cardiomyocytes and PCs were dissociated from *Ncam-1* WT and *Ncam-1* KO crossed with *Cntn2-EGFP* mice. Hearts were Langendorff perfused and enzymatically digested as previously described (Kang et al., 2010). Sorting of myocytes was performed as previously described (Hattori et al., 2010). Briefly, tetramethylrhodamine methyl ester perchlorate (TMRM, Invitrogen) mitochondrial dye was used to identify cardiomyocytes and sort them into RFP^+^ and RFP^−^ populations. Dissociated ventricular cardiomyocytes were incubated with 50 nM TMRM for 15 min and purified using the On-Chip microfluidic cell sorter using a 150µm-wide sorting chip (Nakacho, Japan) into PC (RFP^+^GFP^+^) and VM (RFP^+^GFP^−^) populations. RNA was extracted using the PicoPure RNA Isolation Arcturus Kit.

### RNA extraction, RNA sequencing and quantitative RT-PCR

RNA from adult mouse hearts was extracted from cells captured onto thermoplastic film using the PicoPure RNA Isolation Kit (Arcturus Engineering). For RNA-seq, RNA from sorted PCs and VMs was amplified and libraries prepared using the Automated Nugen Ovation Trio Low input RNA (Nugen). RNA-seq experiments were performed at the NYU School of Medicine Genome Technology Center. Samples were sequenced as 50 bp paired-end reads at 10 million to 20 million reads per replicate on an Illumina HiSeq2500 instrument. All the reads were mapped to the mouse reference genome (mm10) using the STAR aligner (v2.5.0c).(Dobin et al., 2013) Alignments were guided by a Gene Transfer File (GTF, version GRCm38.74) and the mean read insert sizes and their standard deviations were calculated using Picard tools (v.1.126) (http://broadinstitute.github.io/picard/). The read count tables were generated using HTSeq (v0.6.0) (Anders et al., 2015) and normalized based on their library size factors using DESeq2 (v3.0) (Love et al., 2014) and differential expression analysis was performed. The reads per million (RPM) normalized BigWig files were generated using BEDTools (v2.17.0) (Quinlan and Hall, 2010) and bedGraphToBigWig tool (v4), and downstream statistical analyses and plot generation were performed in R environment (v3.1.1) (http://www.r-project.org/). Gene ontology analysis was performed with DAVID (http://david.abcc.ncifcrf.gov/). After analysis, candidate genes were validated with qPCR using primers specific to the gene of interest (Table S1). cDNA was prepared from RNA samples using the Maxima First Strand cDNA Synthesis kit (Thermo Fisher Scientific) and qPCR was performed using the PowerUp SYBR Green kit (Thermo Fisher Scientific) on a StepOne Real-Time PCR System (Applied Biosystems). Primer pairs for qPCR were purchased from Origene when available and used according to the manufacturer's protocol. Additional primer pairs were designed or identified and validated from prior literature.

### Western blot analysis

Whole ventricles and whole brain samples were collected from embryonic (E10.5, E13.5, E16.5, E18.5), postnatal (P1 and P7) and adult mice and immediately cryopreserved in liquid nitrogen. Ventricles were homogenized in RIPA buffer containing protease and phosphatase inhibitors (150 mM NaCl, 1.0% NP-40 or 0.1% Triton X-100, 0.5% sodium deoxycholate, 0.1% sodium dodecyl sulfate, 50 mM Tris-HCl pH 8.0, protease and phosphatase inhibitors). Samples were run on 10% precast polyacrylamide gradient gels (Invitrogen) and transferred to nitrocellulose (Bio-Rad) overnight at 4°C. Nitrocellulose membranes were incubated in blocking buffer consisting of PBS with Tween-20 (0.05%) and 5% nonfat dry milk. Membranes were then incubated with specific primary antibodies diluted in 5% nonfat dry milk overnight at 4°C followed by wash steps and secondary antibodies. Antigen complexes were visualized with the Odyssey Imaging System (LI-COR) and analyzed with Image Studio Lite software.

### Electrocardiograms

To record surface ECGs, mice were anesthetized inhaled 2% isoflurane and subcutaneous electrodes were attached to the four limbs, as previously described (Gutstein et al., 2003). Core body temperature was kept 37.5°C using a heat lamp and the HR monitored throughout. ECG analysis was performed in an unbiased fashion where 100 beats were analyzed using LabChart 7 Pro version 7.3.1 (ADInstruments). Detection and analysis of P wave, PR interval, QRS wave and QT intervals were set to ‘Mouse ECG’ parameters.

### Transthoracic echocardiography

Echocardiography was performed using the Vevo 2100 high-resolution ultrasound imaging system with a real-time 30 MHz linear array scanhead (MS400) at a frame rate of 235 fps, a focal length of 8 mm and a 10×10-mm field of view (VisualSonics, Toronto, Canada). Mice were anesthetized with 2% isoflurane, and hair was removed from the chest using a depilatory cream (Nair; Church & Dwight Co, Princeton, NJ, USA). Warmed ultrasound transmission gel was placed on the chest and used to obtain left ventricular endpoints of cardiac function. B-mode cardiac imaging was conducted on the longitudinal (long-axis) and transverse (short-axis) plane. The aortic valve and apex were used for the long axis imaging landmarks and the papillary muscles were used for the short axis imaging landmark. M-mode recordings of the left ventricle were also recorded at the short axis B-mode imaging plane to obtain left ventricular function and dimensions through the cardiac cycle. HR was monitored, and core body temperature was maintained at 37.5°C using a heated platform and heat lamp throughout the procedure. Mice with HRs below 400 bpm were excluded from the analysis. Data analysis was performed on VisualSonics Vevo 2100 V1.5.0 software (VisualSonics).

### HPS whole-mount quantification

PC imaging and quantification of EGFP fluorescence was conducted using *Cntn2-*EGFP reporter mice. *Ncam-1*, *Cntn2* and *Alcam* mouse lines were crossed with the *Cntn2-*EGFP reporter line to study the spatial and temporal expression of these Ig-SFCAMs within the CCS as reported by EGFP. Hearts were excised and immediately placed in ice-cold PBS and fixed overnight in 4% PFA. For imaging of the left ventricular (LV) HPS, the LV wall was cut open at the center of the free wall. Free wall edges were pinned down using 30-gauge needles to expose the LV septum. For imaging the right ventricular HPS, the anterior portion of the RV free wall adjacent to the septum was cut to expose the RV septum and free wall Purkinje fiber network. Bright-field and fluorescent images of the hearts were taken using the Zeiss M2Bio microscope equipped with a Zeiss AxioCam Color camera interfaced with Zeiss Axiovision 2012 software. Overlay of bright field and fluorescent channels was performed using Photoshop CC 2018. Littermates were imaged on the same day at comparable magnification, exposure and light intensity. All images were processed in MATLAB as follows. First, a morphological top-hat filtering on the grayscale image of the GFP channel was applied using the imtophat function, followed by a binerization of the image using im2bw and graythresh functions. Next, the images were masked according to manually created masks containing the region of interest, and skeletons and branching points were detected using the bwmorph function. Their densities were calculated per unit of masked region. All comparisons between WT and KO groups were made by applying a two-tailed Student's *t*-test.

### Electron microscopy

Anesthetized mice were fixed by cardiac perfusion with freshly prepared solution containing 4% PFA in 0.1 M sodium phosphate buffer, pH 7.3. The heart was dissected and continue fixed at 0.1 M phosphate buffer (PB) containing 2% PFA and 2.5% glutaraldehyde at 4°C overnight. Fixed mouse heart was subsequently processed with modified OTTO (Vanslembrouck et al., 2018) and embedded in Durcupan (Fluka). In brief, the heart tissue was washed with 0.1 M PB, post fixed in 2% OsO_4_ /1.5% potassium ferrocyanide for 1.5 h at room temperature, then stained with freshly made 1% tannic acid (Electron Microscopy Sciences) in PB twice for 2 h each time at 4°C to allow for additional staining. The tissue was then washed in ddH_2_O, placed in 2% aqueous OsO_4_ for 40 min at room temperature, and *en bloc* stained in 1% aqueous uranyl acetate at 4°C overnight. The tissue was then washed with ddH_2_O, dehydrated in a series of ethanol solutions (30, 50, 70, 85, 95, 100, 100%; 10 min each, on ice) and replaced with ice-cold dry acetone for 10 min, followed by 10 min in acetone at room temperature. The sample was gradually equilibrated with Durcupan ACM embedding resin (Electron Microscopy Sciences) and embedded in freshly made 100% Durcupan.

The sample block was trimmed, and thin sections were cut on slot grids to identify the area of interest. The sample block was then mounted on an aluminum specimen pin (Gatan, Pleasanton, CA, USA) using silver conductive epoxy (Ted Pella) to electrically ground the tissue block. The specimen was trimmed again and coated with a thin layer of gold/palladium (Denton Vacuum DESKV sputter coater, Denton Vacuum, NJ, USA). Serial block face imaging was performed using a Gatan OnPoint BSE detector in a Zeiss GEMINI 300 VP FESEM equipped with a Gatan 3View automatic microtome unit. The system was set to cut sections with 75 nm thickness, imaged with gas injection setting at 40% (2.9E-03mBar) with Focus Charge Compensation to reduce the charge, and images were recorded after each round of section from the block face using the SEM beam at 1.5 keV with a dwell time of 1.0 μs/pixel. Each frame is 32Å∼32 μm with pixel size of 2 nm. Data acquisition was automated using Gatan Digital Micrograph (version 3.31) software. A stack of 150 slices was aligned, assembled using ImageJ, with a volume of 32×32×11 µm^3^ dimensions obtained from the tissue block. Segmentation and movies were generated by ORS Dragonfly 4.1.

### Statistics

Endpoints were compared using one-way ANOVA or two-tailed Student's *t*-test where appropriate. *P*<0.05 was considered statistically significant. Sample size calculations were performed using preliminary data to design the experiment for measuring continuous variables. Groups were constructed to detect a 30% difference between experimental and control groups with a power of 90% and a significance level of 0.05. Mean and standard error of the mean are reported for each group. Operators were blinded to experimental groups until the endpoints were analyzed.

### Study approval

All protocols conformed to the Association for the Assessment and Accreditation of Laboratory Animal Care and the NYU School of Medicine Animal Care and Use Committee under protocol IA16-01599.

## Supplementary Material

Supplementary information

Reviewer comments
