# Supplementary material for: Neural cell adhesion molecule is required for ventricular conduction system development
Source: Development. 2021 Jun 7;148(11):dev199431. doi: 10.1242/dev.199431 (PMC8217711; doi:10.1242/dev.199431)
Supplement: Supplementary information [file develop-148-199431-s1.pdf]

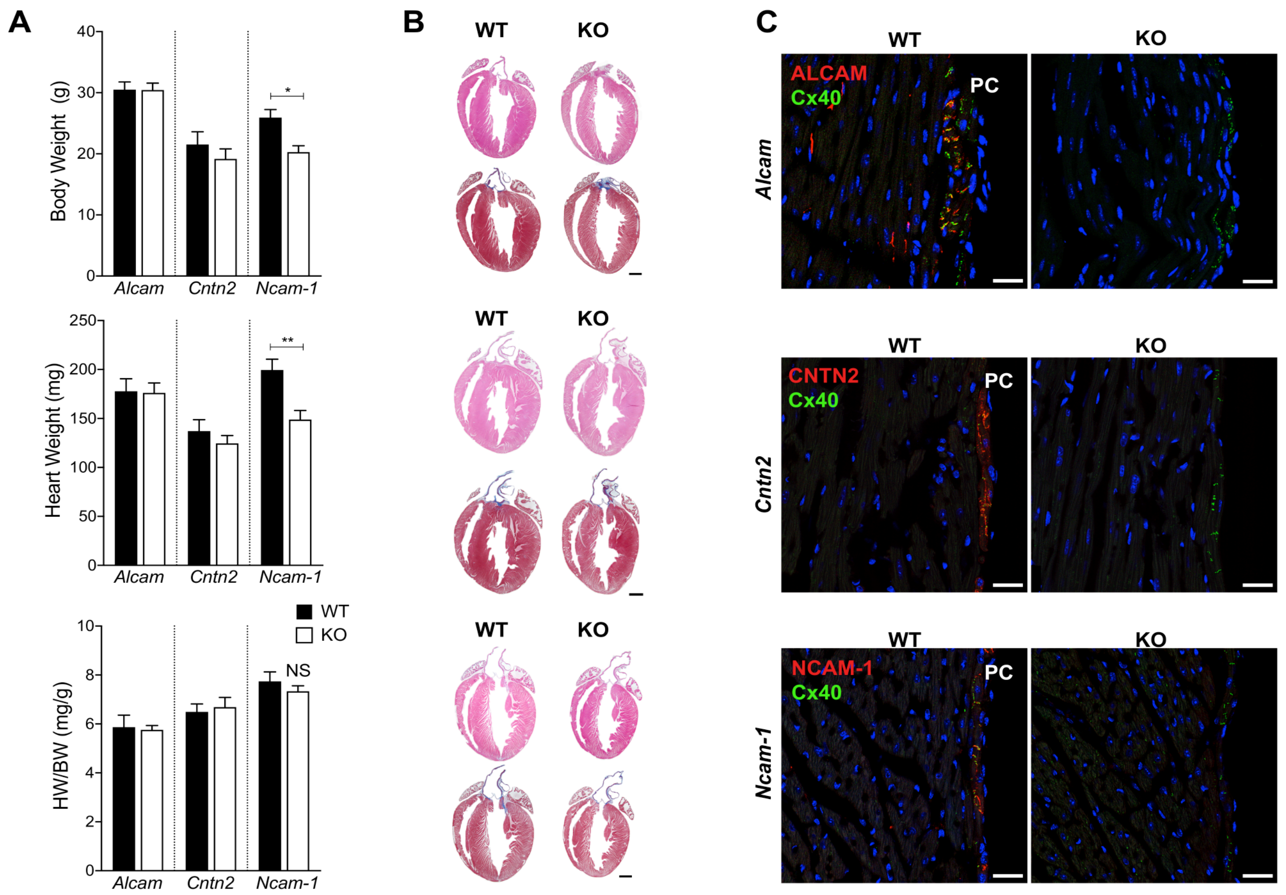

Fig. S1. Structural assessment of Alcam, Cntn2 and Ncam-1 WT and KO hearts. (A) Heart weight, (HW) Body weight, (BW) and HW/BW ratios of Alcam (WT n=6, KO=11), Cntn2 (WT n=8, KO=9) and Ncam-1 (WT n=8, KO=10) mice. Not significant (NS). (B) Hematoxylin and eosin and trichrome stain of Alcam, Cntn2 and Ncam-1 WT and KO hearts. (C) immunofluorescence staining (40x) of Purkinje cells in ventricular myocardium of WT and KO hearts with Cx40 and respective antibody againt each knockout (ALCAM, CNTN2 and NCAM-1). Data represent mean ± SEM. *P<0.05 WT vs KO. **P<0.01 WT vs KO. Scale bar: 25μm (40x images, A-C) and 1mm (whole hearts, A-C).

Development • Supplementary information

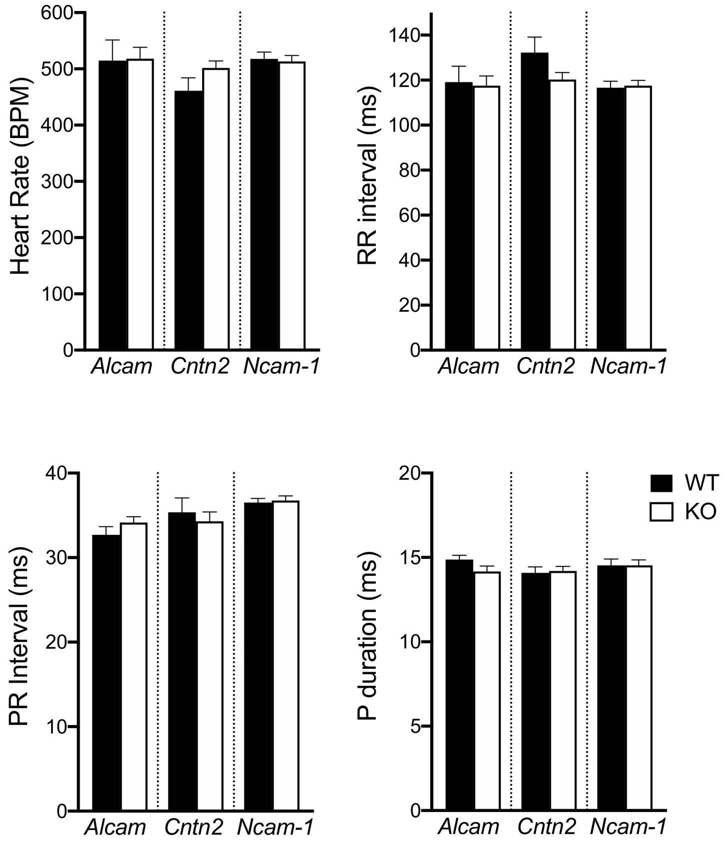

Fig. S2. ALCAM, CNTN2 and NCAM-1 deficient mice display normal HR, P duration, PR internal and RR interval. No difference was detected in Heart rate, P duration, PR interval and RR interval of adult male Cntn2 (WT n=6, KO=8), Alcam (WT n=6, KO=10) and Ncam-1 KO (WT n=11, KO n=12) mice and their littermate WT controls. Data represent mean ± SEM.

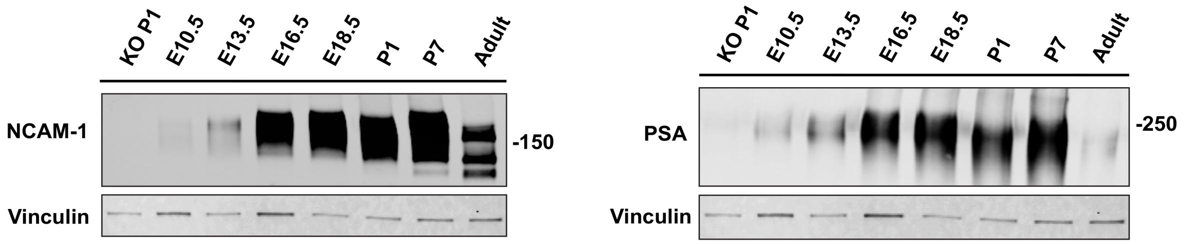

Fig. S3. NCAM-1 and PSA protein expression in whole brain. Western blot analysis of protein extract from whole brain at specified developmental stages using anti-NCAM-1 and anti-PSA-NCAM-1 antibodies. Vinculin was used as loading control. Lysate protein amount loaded: Brain-PSA blot: 1μg, Brain-NCAM-1 blot: 10μg.

Development • Supplementary information

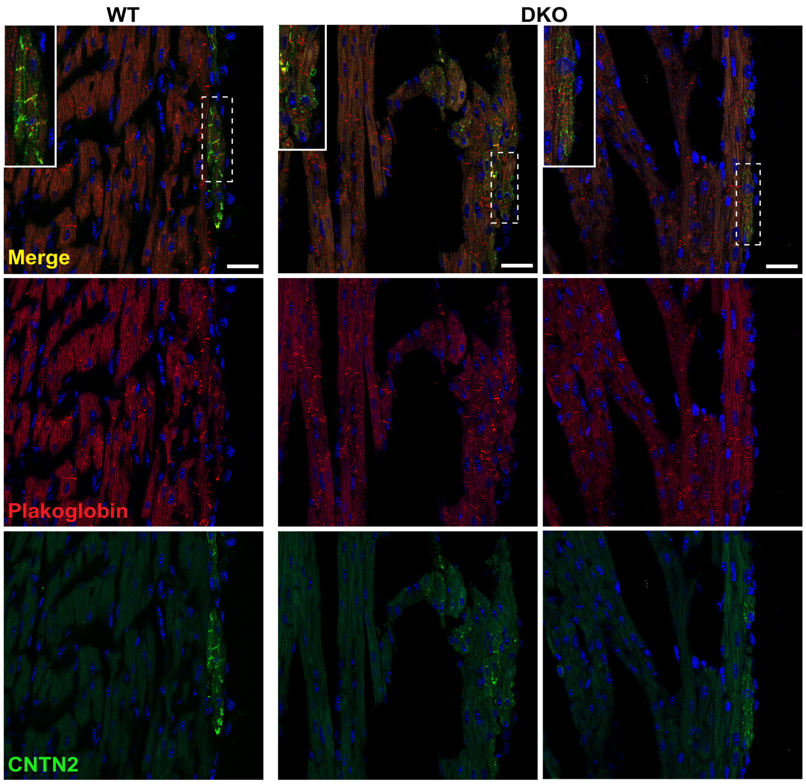

Fig. S4. Mislocalization of cell membrane proteins of Purkinje cells in PSA-deficient mice. Immunofluorescence staining of P21 WT and PSA-deficient hearts sections with antibodies against Plakoglobin (red) and CNTN2 (green) at 40x. Top left inset is a magnified image of dotted white demarcated region. Scale bar: 25μm.

Development • Supplementary information

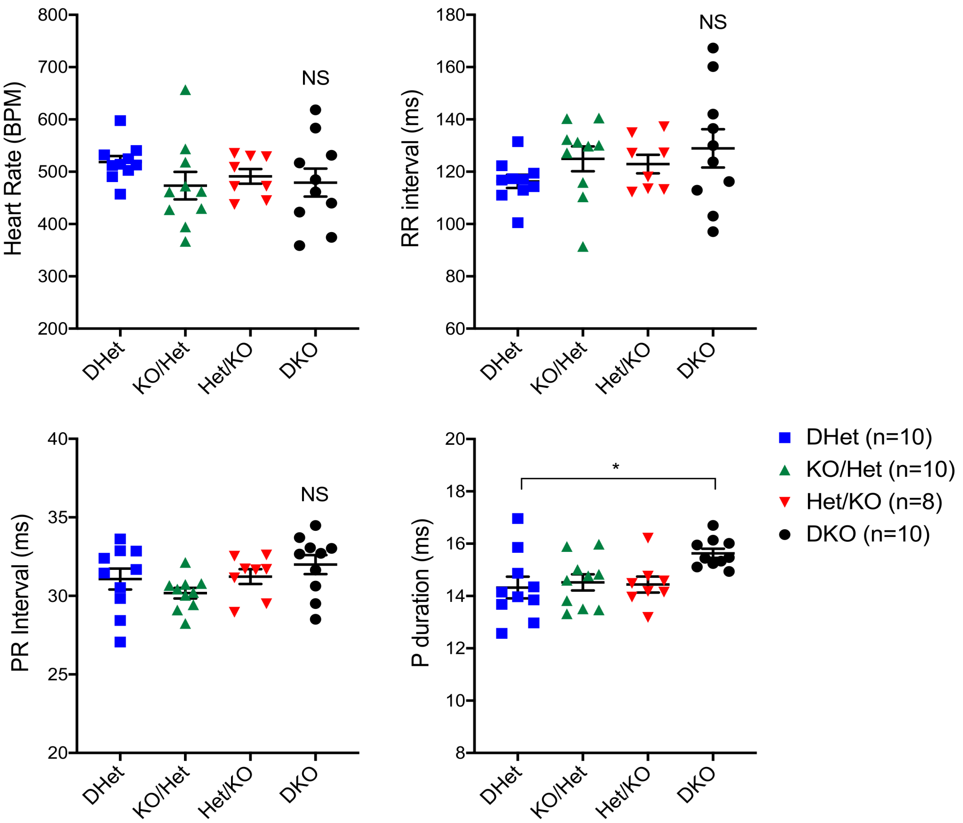

Fig. S5. Electrocardiogram of PSA-deficient DKO mice. Electrocardiograms of PSA-deficient DKO (n=10) mice and littermates with allelic combinations: DHet (n=10), Het/KO (n=10) and KO/Het (n=8). No difference was detected in heart rate, PR interval and RR interval between groups. P-duration was only statistically different between DKO mice and Het/Het littermates. Data represent mean ± SEM. *P<0.05, one-way ANOVA.

Development • Supplementary information

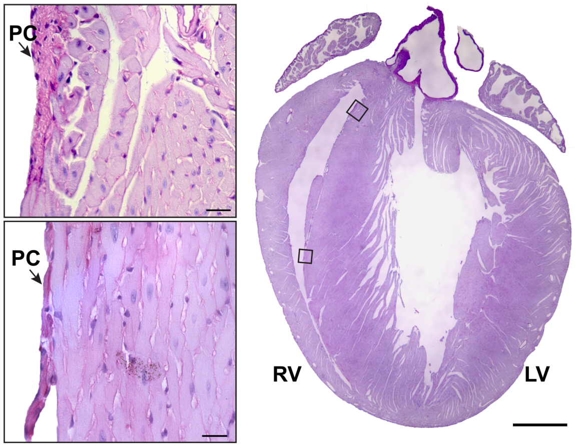

Fig. S6. Glycogen enrichment in murine Purkinje cells. Periodic acid-Schiff (PAS) staining of WT adult heart indicating high glycogen levels in Purkinje cells. High magnification images on the left are from black inset in low magnification whole heart image on the right. Scale bars: 25 μm (left) and 1mm (right).

Table S1. Oligonucleotides for quantitative real time PCR analysis

OriGene Technologies (MP203893): product length = 144
Mouse *Drd2* F 5’- CCTGTCCTTCACCATCTCTTGC-3’
Mouse *Drd2* R 5’- TAGACCAGCAGGGTGACGATGA-3’

OriGene Technologies (MP202908): product length = 128
Mouse *Cntn2* F 5’- CTCCAGCAGAATCCGCACTAAG-3’
Mouse *Cntn2* R 5’- CTCCATTCTGGTACTCTCGTGAC-3’

OriGene Technologies (MP205237) product length = 128
Mouse *Gja5* F 5’- GTGCCAAACCAGGAGCAGATTC-3’
Mouse *Gja5* R 5’- CGCCGTTTGTCACTATGGTAGC-3’

OriGene Technologies (MP215622) product length = 115
Mouse *SCN4B* F 5’- GGCAGATACACCTGCTTCGTGA-3’
Mouse *SCN4B* R 5’- TGAGAGTCACCGTGTTGTCCAC-3’

OriGene Technologies (MP208945) product length = 130
Mouse *Nkx2-5* F 5’- TGCTCTCCTGCTTTCCCAGCC-3’
Mouse *Nkx2-5* R 5’- CTTTGTCCAGCTCCACTGCCTT-3’

OriGene Technologies (MP204358) product length = 127
Mouse *Etv1* F 5’- TCCTGGCTCATCCAAGCAGAAC-3’
Mouse *Etv1* R 5’- CGGTACATTCCAGGCTCTTGCT-3’

Product length = 92
Mouse *Pcp4* F 5’-CGACATGGATGCACCAGAGACAG-3’
Mouse *Pcp4* R 5’-AGGACTGTGATCCTGCCTTTTT-3’

Product length = 240
Mouse *Gapdh* F 5’- TGTCAGCCATGCATCCTGCA-3’
Mouse *Gapdh* R 5’- CCGTTCAGCTCTGGGATGAC-3’

Table S2. Targeted chromosomal mutations in *ST8Sia2* and *ST8Sia4* using CRISPR/Cas9.

ST8sia2	DNA sequence	Mutation
WT	ATGCAGCTGCAGTTCCGGAGCTGGATGCTGGCCGCGCTTACGCTGCTCG	
DKO	ATGCAGCTGCAGTTCCGGAGCTGGATGCTGGCCGCGCT-ACGCTGCTCG	1bp-T-del

ST8sia4	DNA sequence	
WT	ATGCGCTCAATTAGAAAACGGTGGACCATCTGCACTATAAGTCTACTCC	
DKO	ATGCGCTCAATTAGAAAAAACGGTGGACCATCTGCACTATAAGTCTACTCC	2bp-AA-ins

Development • Supplementary information

Table S3. Sequencing primers used to identify *ST8Sia2* and *ST8Sia4* mutations. Primers with an asterisk (*) were primarily used for Sanger sequencing.

Primer	Sequence
ST8Sia4-Fwd	CTGATTCTGGGATCTCGGCTC
ST8Sia4-Rev*	CCCGGCTCTAAGCACACTTC
ST8Sia2-Fwd*	AGCAACCCTTGTCTGTTGCTG
ST8Sia2-Rev	AGGGGAAGAAGAGATCCCGC

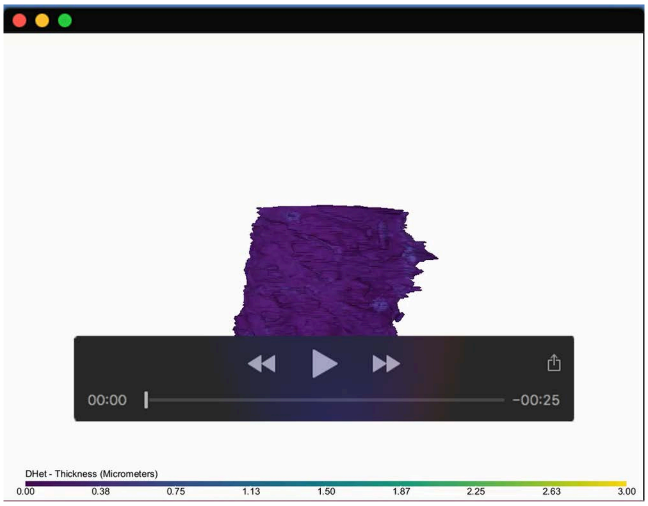

Movie 1. Segmentation video of Ctrl (DHet) mutant mice. Through image acquisition and segmentation, the video shows a reconstruction of the z-stack space between cell junctions of two Purkinje cells.

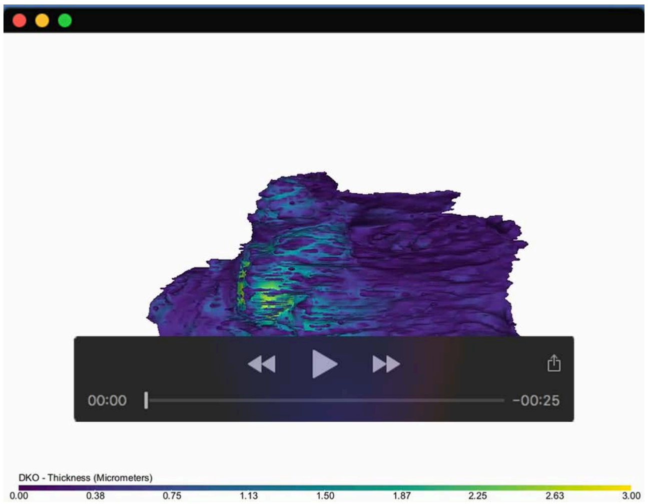

Movie 2. Segmentation video of DKO mutant mice. Through image acquisition and segmentation, the video shows a reconstruction of the z-stack space between cell junctions of two Purkinje cells.

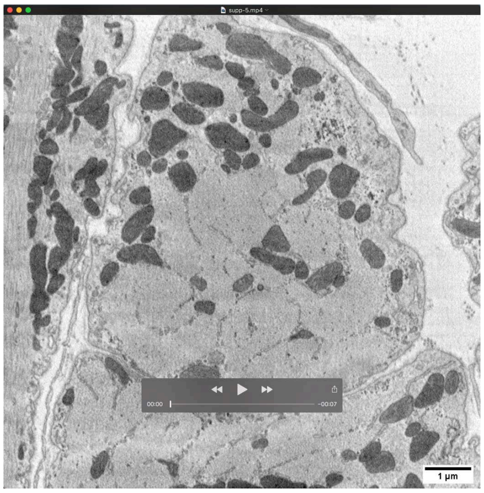

Movie 3. **Z-stack video of Ctrl (DHet) mutant mice, heart 1.** Z-stacks videos of the intercellular space between two additional sets of DHet and DKO Purkinje cell pairs

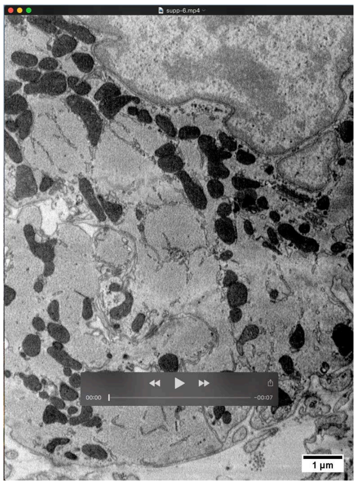

Movie 4. Z-stack video of Ctrl (DHet) mutant mice, heart 2. Z-stacks videos of the intercellular space between two additional sets of DHet and DKO Purkinje cell pairs

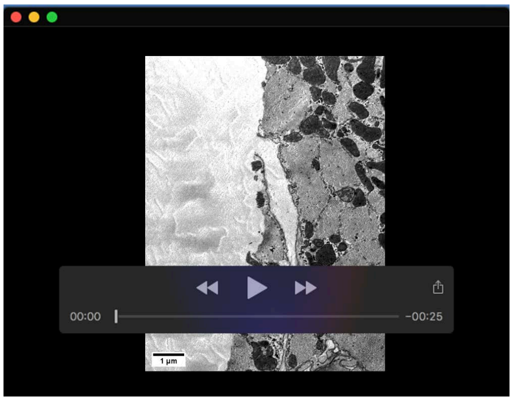

Movie 5. Z-stack video of DKO mutant mice, heart 1. Z-stacks videos of the intercellular space between two additional sets of DHet and DKO Purkinje cell pairs

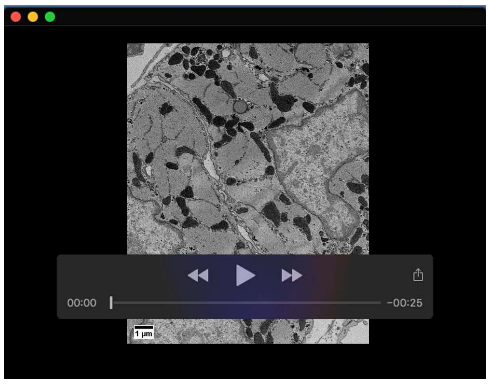

Movie 6. Z-stack video of DKO mutant mice, heart 2. Z-stacks videos of the intercellular space between two additional sets of DHet and DKO Purkinje cell pairs

Movies 1-6. Video data demonstrating expanded extracellular space between apposing Purkinje cells

Development • Supplementary information
