# Supplementary material for: Neural cell adhesion molecule is required for ventricular conduction system development
Source: Development. 2021 Jun 7;148(11):dev199431. doi: 10.1242/dev.199431 (PMC8217711; doi:10.1242/dev.199431)
Supplement: Reviewer comments [file develop-148-199431_review_history.pdf]

Neural cell adhesion molecule is required for ventricular conduction system development

Camila Delgado, Lei Bu, Jie Zhang, Fang-Yu Liu, Joseph Sall, Feng-Xia Liang, Andrew Furley and Glenn I. Fishman
DOI: 10.1242/dev.199431

Editor: Benoit Bruneau

Review timeline

Original submission:	13 January 2021
Editorial decision:	11 March 2021
First revision received:	15 April 2021
Accepted:	26 April 2021

Original submission

First decision letter

MS ID#: DEVELOP/2021/199431

MS TITLE: Neural Cell Adhesion Molecule (NCAM-1) is Required for Ventricular Conduction System Development

AUTHORS: Glenn I Fishman, Camila Delgado, Lei Bu, Jie Zhang, Fang-Yu Liu, Joseph Sall, Feng-Xia Liang, and Andrew Furley

I have now received all the referees reports on the above manuscript, and have reached a decision. The referees' comments are appended below, or you can access them online: please go to BenchPress and click on the 'Manuscripts with Decisions' queue in the Author Area.

The overall evaluation is positive and we would like to publish a revised manuscript in Development, provided that the referees' comments can be satisfactorily addressed. Please attend to all of the reviewers' comments in your revised manuscript and detail them in your point-by-point response. I am not asking for any new experiments, only revisions to the text and/or figures. If you do not agree with any of their criticisms or suggestions explain clearly why this is so.

Reviewer 1

Advance summary and potential significance to field

In this manuscript, Fishman and colleagues present evidence that Ncam-1 is required for normal ventricular conduction system development. Using a previously published gene expression screen, the authors note that several cell adhesion proteins are enriched in the HPS. Temporal expression profiling reveals that Ncam-1 and Alcam are expressed in the HPS, although they are also expressed elsewhere in the heart. Analysis of Cntn2, Ncam-1 and Alcam KO mice reveals a subtle QRS prolongation only in Ncam-1 KO animals, which correlates with reduced numbers and impaired morphogenesis of the HPS. Gene expression profiling uncovers several potential candidates and the HPS stains positive for PSA-NCAM-1. To address the role of Ncam-1 sialylation, the authors generate knockout mice devoid of the enzymatic machinery. These mice share several phenotypic characteristics with Ncam-1 KO mice, including QRS prolongation, suggesting a shared pathway.

© 2021. Published by The Company of Biologists under the terms of the Creative Commons Attribution License (https://creativecommons.org/licenses/by/4.0/).

Furthermore, sialylation negative animals display enlarged intercellular gaps, implying that cell adhesion is impaired. Overall, this is an interesting and well-presented manuscript, and I support publication with a few minor revisions.

Comments for the author

Table 1: Why are the hearts of Ncam-1 KO animals smaller? Is this from decreased proliferation, and, if so, does this relate to the reduction in Cntn2+ HPS cells?

Figure 1: What is Cdh6? Is it also involved at the IC? A discussion of this gene would be helpful.

Figure 2: What about Cx40 expression in 2C? To make the claim that any of the IGsf-CAMs are expressed at the IC, co-localization with N-cad/Cdh2 would strengthen this conclusion. Given the varied spatiotemporal expression patterns, what is the overall conclusion from these studies?

Figure 3: How many mice were included in the analysis for 3A? How many males versus females? For 3B, how many animals were observed to have the morphological phenotype in Ncam-1 KOs (out of the total number analyzed)?

In 3C, is the reduction in GFP+ cells due to increased apoptosis or reduced proliferation? For 3D, the proximo-distal gradient of the Ncam-1 KO phenotype suggests a potential developmental contribution. Have the authors analyzed different developmental time points to see whether HPS ramification is stalled at a particular stage? Since later figures demonstrate mis-localization of Cx40 (7C), it would be worth seeing whether the same holds true in Ncam-1 KO animals.

Furthermore, since the HPS morphological defect in Ncam-1 KO mice does not seem to explain the EKG changes based on the data presented in Figure 7, perhaps the following statement on page 9 should be changed: “From these results, we conclude that abnormalities in Purkinje cell network patterning contribute to the ventricular conduction slowing observed by electrocardiography.”
Figure 4: Based on data presented in later figures, especially the EM data it seems likely that the gene expression changes in this Figure are secondary to structural changes of the HPS cells. Perhaps a re-interpretation of the results from this Figure in the Results (or a more balanced description in the Discussion) is warranted.

Figure 7: For 7F, EMs of Ncam-1 knockouts would be beneficial for comparison/interpretation.

Reviewer 2

Advance summary and potential significance to field

The manuscript by Delgado et al entitled “Neural Cell Adhesion Molecule (NCAM-1) is Required for Ventricular Conduction System Development” identified new cell adhesion molecules highly expressed within the ventricular conduction system. Mice deficient in NCAM-1 exhibit defects in Purkinje cell gene expression, Purkinje patterning as assessed by Cntn2-EGFP, and Purkinje function as assessed by QRS width. Interestingly, the patterning defect appears to be specific to the left side of the heart, while no morphological defects were observed in the right Purkinje system. The authors generated novel knockout mice of enzymes that post-translationally modify NCAM-1 by polysialic acid. Absence of PSA modification also partially phenocopies the NCAM-1 KO mice with respect to functional Purkinje cell disruption (QRS prolongation) without affecting Purkinje patterning.
Interestingly, PSA deficiency results in mis-targeting of multiple sarcolemmal proteins to the intercalated disc. Overall, this manuscript is of high quality and adds significantly to our understanding of the development of the His-Purkinje system, as well as insights into functional conduction defects.

© 2021. Published by The Company of Biologists under the terms of the Creative Commons Attribution License (https://creativecommons.org/licenses/by/4.0/).

Comments for the author

Major:
1. Given that expression of endogenous Cntn2 is down-regulated in the Purkinje cells of Ncam-1 KO mice (Figure 4C), it is possible that the loss of Cntn2-EGFP reflects Cntn2 expression changes and not morphologic defects. The authors should confirm mis-patterning of the left His-Purkinje system in Ncam-1 KO mice using an independent measure.

2. Can the authors comment on whether the QRS prolongation in NCAM-1 KO mice is likely to be completely explained by loss of PSA? This could address the relative importance of form versus function in ventricular conduction defects.

3. The authors should attempt to determine whether transcription is disrupted in the ST8Sia2/ST8Sia4 knockouts. This could be done for just a small subset of genes that are dysregulated in the NCAM-1 KOs, such as ETV1, for example. This could help to differentiate amongst possible mechanisms underlying disrupted gene expression in the NCAM-1 KO mice.

Minor:
Page 12, line 293: “tissue and muscle mass few than 20%” should be “fewer than 20%”

<u>Reviewer 3</u>

Advance summary and potential significance to field

The manuscript by Delgado and colleagues have identified NCLAM-1 (a member of the IgSF-CAM family) as an important player in Purkinje cell development, ventricular conduction patterning and ventricular conduction (QRS interval). In general, we have limited knowledge of the major drivers of ventricular conduction and especially Purkinje development, thus, this manuscript provides insight into new pathways that are important in establishing their role in cardiac development. These pathways will be important to further dissect as they may have significance in identifying biological underpinnings of ventricular arrhythmias that are often fatal, especially in genetic based cardiac diseases. In addition, much of the focus in this field has been on ion channel biology, thus, this manuscript would provide novel insights into biological cell-cell junction mediators of ventricular conduction and disease.

Comments for the author

Overall, this is a very interesting manuscript that brings new knowledge of developmental regulators of the ventricular conduction system, which are lacking in the field. The expression studies are quite elegant and the findings also highlight subpopulation of cells within the ventricular conduction system that are targeted by NCLAM-1, which suggests that the ventricular conduction system is under unique spatial control. In addition, the manuscript provides insight into how post-translational control (polysialyation) of NCLAM-1 may be important in driving Purkinje cell-cell interactions and ventricular conduction. Further comments are provided in order to strengthen the manuscript:

Comments:
1. The authors report QRS prolongation in NCLAM-1 KO mice and DKO mice; however, it is not clear if arrhythmias were found in these mice. The authors should evaluate and discuss if these were evaluated in mice. If they were not observed, could catecholamine stress predispose NCLAM-1 deficient or DKO mice to arrhythmias? This is an important implication of these studies as it could be an important molecular target to assess predisposition to fatal arrhythmias associated with ventricular depolarization delay.

2. The authors should add to their discussion as to potential reasons why the left HPS was primarily impacted by NCLAM-1 KO, as that is a unique feature of their model. Have distinct genes been assigned to the subcompartments of the HPS before?

© 2021. Published by The Company of Biologists under the terms of the Creative Commons Attribution License (https://creativecommons.org/licenses/by/4.0/).

3. There is a bit of a disconnect between the cardiac phenotype from the polysialition NCLAM-1 deficient mice (more severe impact on cardiac mass) and NCLAM-1 deficient mice. The authors state, "mis-targeting phenotype is not observed in NCLAM-1 deficient mice"; however, this data is not shown. Was an impact on loss of cell-cell junction integrity observed in NCLAM-1 deficient mice? In the DKO mice, could other genes be impacted in the polysialition deficient settings? The manuscript could benefit from a streamlined discussion on the distinct and parallel phenotypes observed between both models to provide clarity on the mechanisms underlying NCLAM-1's role and its posttranslational modifications in driving ventricular conduction development and disease.

First revision

Author response to reviewers' comments

Reviewer 1

We thank the reviewer for characterizing our manuscript as interested and well-presented. Below we address each of your comments.

Table 1: Why are the hearts of Ncam-1 KO animals smaller? Is this from decreased proliferation, and, if so, does this relate to the reduction in Cntn2+ HPS cells?

Ncam-1 mutant mice were originally generated by Cremer et al (Cremer et al, *Nature*, 1994) and homozygous mutants were reported to weigh about 10% less than wild-type and heterozygous littermates. In the Cremer paper, which focused on brain development, they reported that even after correction for the reduced body weight, the olfactory bulb was 36% smaller than wildtypes and heterozygotes and the reduction in the olfactory bulb was mainly a consequence of the smaller size of the granular cell layer, an area showing the strongest expression of NCAM. The mechanisms responsible for the modest decrease in the overall weight of the animals has not been reported. There is evidence that nervous system neurotransmitter pathways that influence body weight may be influenced by molecules regulating cell adhesion, such as syndecan-3 (Reizes et al, *Int J Biochem Cell Biol*, 2007), however whether or not this logic extends to additional cell adhesion proteins such as NCAM1 is speculative. In our experiments, we observed the same ~10% reduction in overall weight of the NCAM1 KO mice, and overall the heart weight to body weight ratio was preserved, meaning that the heart itself was proportionately sized for the overall size of the mouse. Taken together, we believe that NCAM1 mutant mice have a generalized runting phenotype (the 10% reduction in virtually all organs) as well as a specific cell-autonomous deficit in cell types in which NCAM1 and/or PSA-NCAM1 are highly expressed, such as the olfactory bulb and ventricular conduction system.

- Cremer, H., Lange, R., Christoph, A. *et al.* Inactivation of the N-CAM gene in mice results in size reduction of the olfactory bulb and deficits in spatial learning. *Nature* 367, 455–459 (1994).
- Reizes O, Benoit SC, Clegg DJ. The role of syndecans in the regulation of body weight and synaptic plasticity. *Int J Biochem Cell Biol* 40(1):28-45 (2008).

Figure 1: What is Cdh6? Is it also involved at the IC? A discussion of this gene would be helpful.

Cdh6 encodes cadherin-6, a membrane glycoprotein that mediates homophilic cell-cell adhesion (Shimoyama et al, *Cancer Res*, 1995). It is been used as a marker of neural crest cells in prior developmental studies (Ishii et al, *Development*, 2005). There are no published data I am aware of implicating Cdh6 expression in intercalated disc function. Cdh6 was included in Figure 1A because of its substantial enrichment in Purkinje cells and its function in cell adhesion. Inasmuch as the manuscript focuses on a specific class of cell adhesion proteins (immunoglobulin super-family), we did not further characterize its biology, however we agree with the reviewer that further mention of this gene would be a useful addition to the manuscript and have included

© 2021. Published by The Company of Biologists under the terms of the Creative Commons Attribution License (https://creativecommons.org/licenses/by/4.0/).

such text in the revised Discussion (lines 494-500).

- Shimoyama Y, Gotoh M, Terasaki T, Kitajima M, Hirohashi S. Isolation and sequence analysis of human cadherin-6 complementary DNA for the full coding sequence and its expression in human carcinoma cells. *Cancer Res*. 55 (10): 2206–11. (1995).
- Ishii M, Han J, Yen HY, Sucov HM, Chai Y, Maxson RE Jr. Combined deficiencies of Msx1 and Msx2 cause impaired patterning and survival of the cranial neural crest. *Development* 132(22):4937-50. (2005).

Figure 2: What about Cx40 expression in 2C? To make the claim that any of the IGsf-CAMs are expressed at the IC, co-localization with N-cad/Cdh2 would strengthen this conclusion. Given the varied spatiotemporal expression patterns, what is the overall conclusion from these studies?

Colocalization of NCAM1 with the intercalated disc protein Cx40 at several stages in the developing heart is presented in Figure 2A and in the postnatal day 21 ventricular conduction (His bundle and Purkinje cells) in Figure 1C (lines 113-115). Together, these images support the conclusion that NCAM1 is indeed co-localized at the intercalated disc and it becomes restricted to the ventricular conduction system as hearts mature.

Figure 3: How many mice were included in the analysis for 3A? How many males versus females?

We performed ECGs on 12 mice for each genotype. We have clarified this in the figure legend. The sex distribution was as follows

Cntn2:	WT = 6M, KO = 8M
ALCAM:	WT = 6M, KO = 10M
NCAM:	WT = 11M, KO = 12M

For 3B, how many animals were observed to have the morphological phenotype in Ncam-1 KOs (out of the total number analyzed)?

We examined 9 NCAM1 knockout mice and 6 were judged to display an overt morphological phenotype in the left HPS. We have included these numbers in the revision (lines 996-997).

In 3C, is the reduction in GFP+ cells due to increased apoptosis or reduced proliferation?

This is an interesting question. In hippocampal progenitor cells there is evidence that NCAM inhibitis proliferation and promotes neural differentiation (Amoureux et al, *J. Neurosci*, 2000; Shin et al, *Exp Mol Med*, 2002). By analogy, we hypothesize that loss of function of NCAM in the developing heart might impair the differentiation of Purkinje cell precursors in the trabecular myocardium. This hypothesis is consistent with our data showing marked downregulation of numerous Purkinje cell enriched transcripts in NCAM1 KO Purkinje cells. In other words, they appear to have a defect in differentiation. We performed Ki67 staining in developing wildtype and NCAM mutant hearts but did not observe a significant difference. We mention this negative result in the revision (lines 180-184). However, given that VCS progenitors are a rather small sub-population of cells in the developing heart and the phenotype is restricted to the mid and apical left VCS, we may not have had adequate sensitivity to detect an imbalance between proliferation and differentiation in our model system.

- Amoureux MC, Cunningham BA, Edelman GM, Crossin KL. N-CAM binding inhibits the proliferation of hippocampal progenitor cells and promotes their differentiation to a neuronal phenotype. *J Neurosci.* 20(10):3631-40.(2000).
- Shin MH, Lee EG, Lee SH, Lee YS, Son H. Neural cell adhesion molecule (NCAM) promotes the differentiation of hippocampal precursor cells to a neuronal lineage, especially to a glutamatergic neural cell type. *Exp Mol Med* 31;34(6):401-10. (2002).

For 3D, the proximo-distal gradient of the Ncam-1 KO phenotype suggests a potential developmental contribution. Have the authors analyzed different developmental time points

© 2021. Published by The Company of Biologists under the terms of the Creative Commons Attribution License (https://creativecommons.org/licenses/by/4.0/).

to see whether HPS ramification is stalled at a particular stage? Since later figures demonstrate mis-localization of Cx40 (7C), it would be worth seeing whether the same holds true in Ncam-1 KO animals.

We agree with the reviewer that the abnormal patterning of the VCS in *Ncam-1* KO mice almost certainly has a development contribution. The Cntn2-EGFP reporter gene does not become expressed to any appreciable degree until after birth, and therefore we could not perform the whole-mount visualization of the His-Purkinje system during early development time points. With respect to Cx40 mislocalization, this phenotype was observed in the polysialylation-deficient DKO mice (Figure 7C) but was not seen in the Ncam-1 KO mice. This result is shown in revised Figure S1 and mentioned in the revised text (lines 147-149).

Furthermore, since the HPS morphological defect in Ncam-1 KO mice does not seem to explain the EKG changes based on the data presented in Figure 7, perhaps the following statement on page 9 should be changed: "From these results, we conclude that abnormalities in Purkinje cell network patterning contribute to the ventricular conduction slowing observed by electrocardiography."

The data presented in Figure 3 suggest that a morphological defect in the patterning of the left VCS may play a mechanistic role in the ECG changes (QRS prolongation) recorded in NCAM1 KO mice. The data presented in Figure 7 are from the polysialylation deficient DKO mice, which do not have the overt VCS patterning defect but do have ultrastructural and protein trafficking defects. Given the abnormalities in Purkinje cell gene expression in NCAM1 KO mutant mice, it is certainly possible that in addition to abnormal patterning of the left-sided VCS, Purkinje cell electrophysiological defects (ion channels, gap junctions, etc.) may also contribute to conduction slowing. We have revised the text to reflect this additional factor (lines 220-222).

Figure 4: Based on data presented in later figures, especially the EM data, it seems likely that the gene expression changes in this Figure are secondary to structural changes of the HPS cells. Perhaps a re- interpretation of the results from this Figure in the Results (or a more balanced description in the Discussion) is warranted.

We agree with the reviewer that loss of function of NCAM1 may very well influence gene expression through multiple mechanisms, including those arising from structural changes in HPS cells. We have revised the Discussion to provide a more balanced presentation of this possibility (lines 418-421).

Figure 7: For 7F, EMs of Ncam-1 knockouts would be beneficial for comparison/interpretation.

As noted in our cover letter to the Handling Editor, during the past year the COVID pandemic here in New York City required us to limit the density of pesonnel in our research labs and severely cull back our mouse colonies. As a result it would take us an inordinate amount of time to generate any new animals for additional experimentation. We agree that additional EMs of NCAM1 knockout mice would be informative and hope to perform such experiments in a subsequent study.

Reviewer 2

We thank the reviewer for characterizing our manuscript as high quality and remarking that it adds significantly to our understanding of the development of the His-Purkinje system, as well as insights into functional conduction defects. Below we address each of your comments.

© 2021. Published by The Company of Biologists under the terms of the Creative Commons Attribution License (https://creativecommons.org/licenses/by/4.0/).

1. Given that expression of endogenous Cntn2 is down-regulated in the Purkinje cells of Ncam-1 KO mice (Figure 4C), it is possible that the loss of Cntn2-EGFP reflects Cntn2 expression changes and not morphologic defects. The authors should confirm mis-patterning of the left His-Purkinje system in Ncam- 1 KO mice using an independent measure.

The reviewer asks whether the abnormal patterning of the left His-Purkinje system really just reflects downregulation of Cnt2-EGFP signal - in other words - the VCS may appear hypoplastic because the fluorescence signal is weaker. Given the observation that the morphogical defect is regional (prominent in the left His-Purkinje system but not right) and that it is associated with a significant (24%) reduction in the number of Purkinje cells as quantified by FACS, it seems unlikely to us that the VCS morphological defect can be attributed solely to down-regulation of the Cntn2-EGFP reporter gene. However we agree that this visual analysis is not disposative. To address this question using another approach, we generated a histogram of EGFP fluorescence intensity versus cell number, reasoning that if down-regulation of the Cntn2-EGFP reporter gene was responsible for the abnormal appearance of the VCS, then the distribution of fluorescence intensity in the KO Purkinje cells should be shifted to the left (weaker fluorescence). As shown to the right, we did not observe such an effect, supporting our original interpretation of the data. Introducing a different reporter gene, such as a Cx40-EGFP reporter (Miquerol et al, *Cardiovas Res*, 2004), would provide an additional independent measure (discounting for the moment the multiple generations of breeding that would be required to generate compound mutant NCAM1 null, Cx40-EGFP reporter mice). However, since our RNA-Seq studies demonstrate that Cx40 expression is also reduced in the NCAM1 knockout mice, the same concerns the reviewer raises with the Cntn2-EGFP reporter would be operative.

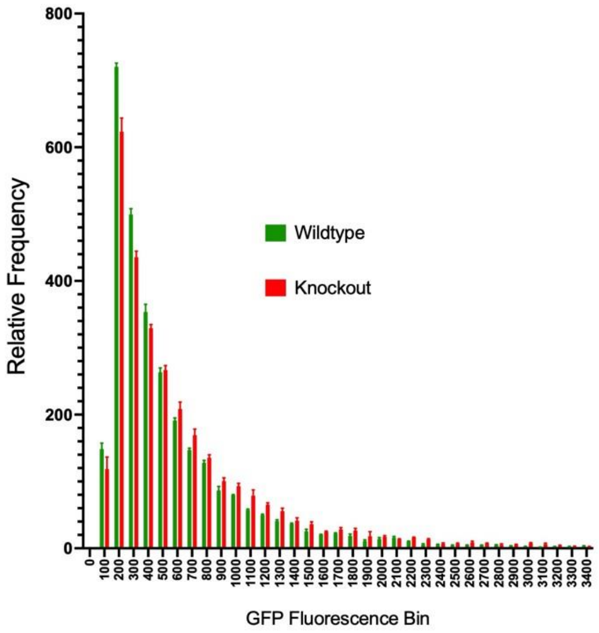

- Miquerol L, Meysen S, Mangoni M, Bois P, van Rijen HV, Abran P, Jongsma H, Nargeot J, Gros D. Architectural and functional asymmetry of the His-Purkinje system of the murine heart. *Cardiovasc Res* 63(1):77-86. (2004).

2. Can the authors comment on whether the QRS prolongation in NCAM-1 KO mice is likely to be completely explained by loss of PSA? This could address the relative importance of form versus function in ventricular conduction defects.

We observed QRS prolongation in both the NCAM1 KO mice as well as the ST8Sia2$^{-/-}$ ST8Sia4$^{-/-}$ double- knockout (DKO) mice. Our data demonstrate abnormal patterning of the VCS in NCAM1 KO mice and ultrastructural defects and aberrant trafficking of junctional proteins in the VCS in DKO mice. While our data allow us to conclude that loss of PSA itself is *sufficient* to cause QRS prolongation, the nature of the two genetic models we employed do not allow us to unequivocally conclude that the QRS prolongation in NCAM1 KO mice is solely attributable to loss of PSA, since both PSA *and* NCAM1 are absent in NCAM deficient mice. In fact, the observation that NCAM1 KO mice have defects in VCS patterning *not observed* in the DKO mice leads us to the opposite conclusion, that loss of function of NCAM1 and loss of post-translational modification of NCAM1 by PSA each confer conduction defects through distinct mechanisms.

3. The authors should attempt to determine whether transcription is disrupted in the

© 2021. Published by The Company of Biologists under the terms of the Creative Commons Attribution License (https://creativecommons.org/licenses/by/4.0/).

ST8Sia2/ST8Sia4 knockouts. This could be done for just a small subset of genes that are dysregulated in the NCAM-1 KOs, such as ETV1, for example. This could help to differentiate amongst possible mechanisms underlying disrupted gene expression in the NCAM-1 KO mice.

This is an excellent suggestion and an important experiment. Unfortunately, as noted elsewhere, all of our mouse colonies were severely culled back in response to the COVID pandemic and the mandated partial closure of our research labs during this past year here in New York City. It would take us many months of breeding to obtain the requisite double knockout mice and obtain mice for this experiment. Accordingly, we don't realistically believe we could complete these studies in a timely fashion for this initial paper. We plan to address this question in a subsequent study designed to further distinguish NCAM deficiency versus PSA deficiency.

Minor:
Page 12, line 293: "tissue and muscle mass few than 20%" should be "fewer than 20%"
Thank you for noting this mistake. We have corrected the text.

Reviewer 3

We thank the reviewer for characterizing our study as a "very interesting manuscript that brings new knowledge of developmental regulators of the ventricular conduction system." Below we address each of your comments.

1. The authors report QRS prolongation in NCLAM-1 KO mice and DKO mice; however, it is not clear if arrhythmias were found in these mice. The authors should evaluate and discuss if these were evaluated in mice. If they were not observed, could catecholamine stress predispose NCLAM-1 deficient or DKO mice to arrhythmias? This is an important implication of these studies as it could be an important molecular target to assess predisposition to fatal arrhythmias associated with ventricular depolarization delay.

We did not observe arrhythmias during the baseline ECG recordings in any of the mutant models investigated in this study. We agree that provocative testing, for example with catecholaminergic stress, such as the caffeine and epinephrine cocktail that we and others have used in the past would be of interest with respect to implicating arrhythmia molecular targets. Unfortunately, as noted in our cover letter to Dr. Bruneau, all of our mouse colonies were severely culled back in response to the COVID pandemic and the partial closure of our research labs during this past year here in NYC. Therefore we cannot realistically perform such a experiment in a timely fashion for this initial paper but hope to examine this important translational question in a subsequent study.

2. The authors should add to their discussion as to potential reasons why the left HPS was primarily impacted by NCLAM-1 KO, as that is a unique feature of their model. Have distinct genes been assigned to the subcompartments of the HPS before?

The reviewer raises an interesting question regarding the differing effects of loss of function of NCAM1 in the left vs. right His-Purkinje system. We have no specific data from our studies providing a mechanistic explanation for the finding. As the reviewer is undoubtedly aware, there are numerous well-characterized developmental and transcriptional programs that are differentially expressed in the left vs right chambers of the heart, for example the HAND genes (Thomas et al, *Dev Biol*, 1998). Morever, the sidedness of the phenotype could conceivably reflect the differential contribution of first versus second heart field precursors to VCS within the left vs right ventricular chambers (Mohan et al, JCDD, 2017). With respect to distinct genes in subcompartments of the HPS, the Dobrzynski group (Atkinson et al, *JMCC*, 2011) has performed qPCR analysis of micro-dissected left versus right free-running Purkinje fibers in rabbits and

© 2021. Published by The Company of Biologists under the terms of the Creative Commons Attribution License (https://creativecommons.org/licenses/by/4.0/).

identified a number of differentially expressed transcripts, including the sodium channel subunits NaV1.5 and Navβ1 (left PF > right PF). In addition, there are some relatively recent and quite elegant single cell profiling data on transcriptional diversity in the developing conduction system from the Wu lab, which reported unique transcriptional signatures for conduction cells within the bundle of His vs. immature Purkinje cells vs. so-called transitional Purkinje cells at the Purkinje-myocyte junction (Goodyer et al, *Circ Res*, 2019). Without being overly speculative, we have added these potential concepts to the Discussion (lines 397- 404).

- Thomas T, Yamagishi H, Overbeek PA, Olson EN, Srivastava D. The bHLH factors, dHAND and eHAND, specify pulmonary and systemic cardiac ventricles independent of left-right sidedness. Dev Biol. 196(2):228-36. (1998)
- Mohan R, Boukens BJ, Christoffels VM. Lineages of the Cardiac Conduction System. *J Cardiovasc Dev Dis.* 4(2):5 (2017).
- Atkinson A, Inada S, Li J, Tellez JO, Yanni J, Sleiman R, Allah EA, Anderson RH, Zhang H, Boyett MR, Dobrzynski H. Anatomical and molecular mapping of the left and right ventricular His-Purkinje conduction networks. *J Mol Cell Cardiol.* 51(5):689-701. (2011).
- Goodyer WR, Beyersdorf BM, Paik DT, Tian L, Li G, Buikema JW, Chirikian O, Choi S, Venkatraman S, Adams EL, Tessier-Lavigne M, Wu JC, Wu SM. Transcriptomic Profiling of the Developing Cardiac Conduction System at Single-Cell Resolution. *Circ Res.* 125(4):379-397 (2019).

3. There is a bit of a disconnect between the cardiac phenotype from the polysialition NCLAM-1 deficient mice (more severe impact on cardiac mass) and NCLAM-1 deficient mice. The authors state, "mis-targeting phenotype is not observed in NCLAM-1 deficient mice"; however, this data is not shown.

As the reviewer notes, the polysialylation deficient DKO mice show a mis-targeting phenotype, whereby several proteins do not appropriately target to the intercalated disc. In contrast, the Ncam-1 KO mice do not appear to show this phenotype. The appropriate targeting of the gap junction protein Cx40 to the intercalated disc of Purkinje cells is shown in a revised Figure S1.

Was an impact on loss of cell-cell junction integrity observed in NCLAM-1 deficient mice? In the DKO mice, could other genes be impacted in the polysialition deficient settings? The manuscript could benefit from a streamlined discussion on the distinct and parallel phenotypes observed between both models to provide clarity on the mechanisms underlying NCLAM-1's role and its posttranslational modifications in driving ventricular conduction development and disease.

As noted above (reviewer 2), while both loss of function of NCAM1 and loss of function of the polysialylation enzymes both lead to conduction abnormalities as evidenced by QRS prolongation, the mechanisms responsible for the convergent conduction phenotype appear to be somewhat distinct. Our data demonstrate abnormal patterning of the VCS in *Ncam-1* KO mice, which is not seen in the DKO mutant mice, and aberrant trafficking of junctional proteins in the VCS in DKO mice, a phenotype not seen in the NCAM1 KO mice.
While these data allow us to conclude that loss of PSA alone is *sufficient* to cause QRS prolongation, the nature of the two genetic models we employed do not allow us to unequivocally conclude that that the QRS prolongation in NCAM1 KO mice is also solely attributable to loss of PSA, since of course both PSA *and* NCAM1 are absent in NCAM deficient mice. In fact, the observation that NCAM1 KO mice have defects in VCS patterning *not observed* in the DKO mice leads us to conclude the opposite, that loss of function of NCAM1 and loss of post-translational modification of NCAM1 by PSA each confer conduction defects through distinct mechanisms. We have attempted to strengthen the discussion of these distinct phenotypes in the revised manuscript.

<u>Second decision letter</u>

MS ID#: DEVELOP/2021/199431

© 2021. Published by The Company of Biologists under the terms of the Creative Commons Attribution License (https://creativecommons.org/licenses/by/4.0/).

MS TITLE: Neural Cell Adhesion Molecule (NCAM-1) is Required for Ventricular Conduction System Development

AUTHORS: Glenn I Fishman, Camila Delgado, Lei Bu, Jie Zhang, Fang-Yu Liu, Joseph Sall, Feng-Xia Liang, and Andrew Furley

ARTICLE TYPE: Research Article

I am happy to tell you that your manuscript has been accepted for publication in Development, pending our standard ethics checks.

Reviewer 1

Advance summary and potential significance to field

In this manuscript, Fishman and colleagues present evidence that Ncam-1 is required for normal ventricular conduction system development. Using a previously published gene expression screen, the authors note that several cell adhesion proteins are enriched in the HPS. Temporal expression profiling reveals that Ncam-1 and Alcam are expressed in the HPS, although they are also expressed elsewhere in the heart. Analysis of Cntn2, Ncam-1 and Alcam KO mice reveals a subtle QRS prolongation only in Ncam-1 KO animals, which correlates with reduced numbers and impaired morphogenesis of the HPS. Gene expression profiling uncovers several potential candidates and the HPS stains positive for PSA-NCAM-1. To address the role of Ncam-1 sialylation, the authors generate knockout mice devoid of the enzymatic machinery. These mice share several phenotypic characteristics with Ncam-1 KO mice, including QRS prolongation, suggesting a shared pathway. Furthermore, sialylation negative animals display enlarged intercellular gaps, implying that cell adhesion is impaired. Overall, this is an interesting and well-presented manuscript, and I support publication.

Comments for the author

The authors have satisfactorily addressed by comments.

Reviewer 2

Advance summary and potential significance to field

This manuscript will significantly add to our understanding of pathways regulating Purkinje cell development and post-natal function.

Comments for the author

The authors have adequately addressed my previous questions and concerns.

Reviewer 3

Advance summary and potential significance to field

The authors have provided new molecular insights into cell adhesion molecules as new drivers of ventricular conduction, which have largely remained elusive to the field. This could have broad implications in better understanding the underlying etiology of ventricular arrhythmias and sudden death in the field.

Comments for the author

I have no further comments.

© 2021. Published by The Company of Biologists under the terms of the Creative Commons Attribution License (https://creativecommons.org/licenses/by/4.0/).
